# Molecular Mechanisms of Western Diet-Induced Obesity and Obesity-Related Carcinogenesis—A Narrative Review

**DOI:** 10.3390/metabo13050675

**Published:** 2023-05-21

**Authors:** Dhruvi Lathigara, Devesh Kaushal, Robert Beaumont Wilson

**Affiliations:** 1 Department General Surgery, UWS, Campbelltown Hospital, Campbelltown, NSW 2560, Australia; 2 Department Upper Gastrointestinal Surgery, UNSW, Liverpool Hospital, Liverpool, NSW 2170, Australia

**Keywords:** bariatric surgery, breast cancer, colorectal carcinoma, crown-like structure (CLS), cytokine, exosome, GLP-1, HIF-1α, hypoxia, leptin, macrophage polarization, metabolic syndrome, NASH, obesity, senescence

## Abstract

The present study aims to provide a narrative review of the molecular mechanisms of Western diet-induced obesity and obesity-related carcinogenesis. A literature search of the Cochrane Library, Embase and Pubmed databases, Google Scholar and the grey literature was conducted. Most of the molecular mechanisms that induce obesity are also involved in the twelve Hallmarks of Cancer, with the fundamental process being the consumption of a highly processed, energy-dense diet and the deposition of fat in white adipose tissue and the liver. The generation of crown-like structures, with macrophages surrounding senescent or necrotic adipocytes or hepatocytes, leads to a perpetual state of chronic inflammation, oxidative stress, hyperinsulinaemia, aromatase activity, activation of oncogenic pathways and loss of normal homeostasis. Metabolic reprogramming, epithelial mesenchymal transition, HIF-1α signalling, angiogenesis and loss of normal host immune-surveillance are particularly important. Obesity-associated carcinogenesis is closely related to metabolic syndrome, hypoxia, visceral adipose tissue dysfunction, oestrogen synthesis and detrimental cytokine, adipokine and exosomal miRNA release. This is particularly important in the pathogenesis of oestrogen-sensitive cancers, including breast, endometrial, ovarian and thyroid cancer, but also ‘non-hormonal’ obesity-associated cancers such as cardio-oesophageal, colorectal, renal, pancreatic, gallbladder and hepatocellular adenocarcinoma. Effective weight loss interventions may improve the future incidence of overall and obesity-associated cancer.

## 1. Introduction

There are a number of recent developments in the epidemiology, pathophysiology, investigation and management of obesity. It has been recognized that patients who are obese are substantially more likely to have a shortened lifespan compared to patients of lean weight, due to obesity-related co-morbidity, metabolic syndrome and an increased risk of cancer. Human obesity involves behavioural, psychosocial, neuroendocrine, immunological, dietary, gut microbiome, genetic and epigenetic factors. These are intertwined with major changes in global food production and distribution, as well as decreased physical activity in an increasingly urbanized society [1,2,3,4,5,6,7,8,9,10,11,12,13,14,15,16,17,18]. The aim of this narrative review was to examine:

1. The current global phenomenon of excess body weight.
2. Excess energy intake and inadequate energy expenditure in obesity.
3. The ‘thrifty gene’ versus the ‘thrifty epigenome’ hypothesis.
4. Cell-to-cell communication between adipose tissue and other organs in the development of obesity and metabolic syndrome.
5. The role of Western diets, fat and carbohydrate metabolism in obesity and obesity-related carcinogenesis.
6. The molecular mechanisms of obesity and relationship with the 14 Hallmarks of Cancer and enabling characteristics.
7. Weight loss interventions in the resolution of metabolic syndrome, adipose tissue dysfunction and prevention of future cancers in patients with obesity.

## 2. Methods

A literature search from 2007–2023 was performed, including the Cochrane Library, Embase and Pubmed databases, Google Scholar and the grey literature. Additional studies were sourced from the reference lists of relevant studies. The initial search was limited to studies in English from 2007 to the present.

## 3. Discussion

### 3.1. Epidemiology of Obesity

In 1997, the World Health Organization (WHO) recognized obesity as a serious, complex chronic disease, and one that was not just restricted to affluent Western nations. It involved all age groups, socio-economic classes and industrialized and developing countries and was indeed a global epidemic [1,2,3,4,5,6,7,8,9,10,11,12,13,14,15,16]. The WHO definitions of excess body weight in adults based on body mass index (BMI) are:

Overweight (BMI = 25–29.9 kg/m^2^).
Obese (BMI ≥ 30 kg/m^2^).
Class I obesity (BMI = 30–34.9 kg/m^2^),
Class II obesity (BMI= 35–39.9 kg/m^2^),
Class III (BMI ≥ 40 kg/m^2^).

**Figure 1:**
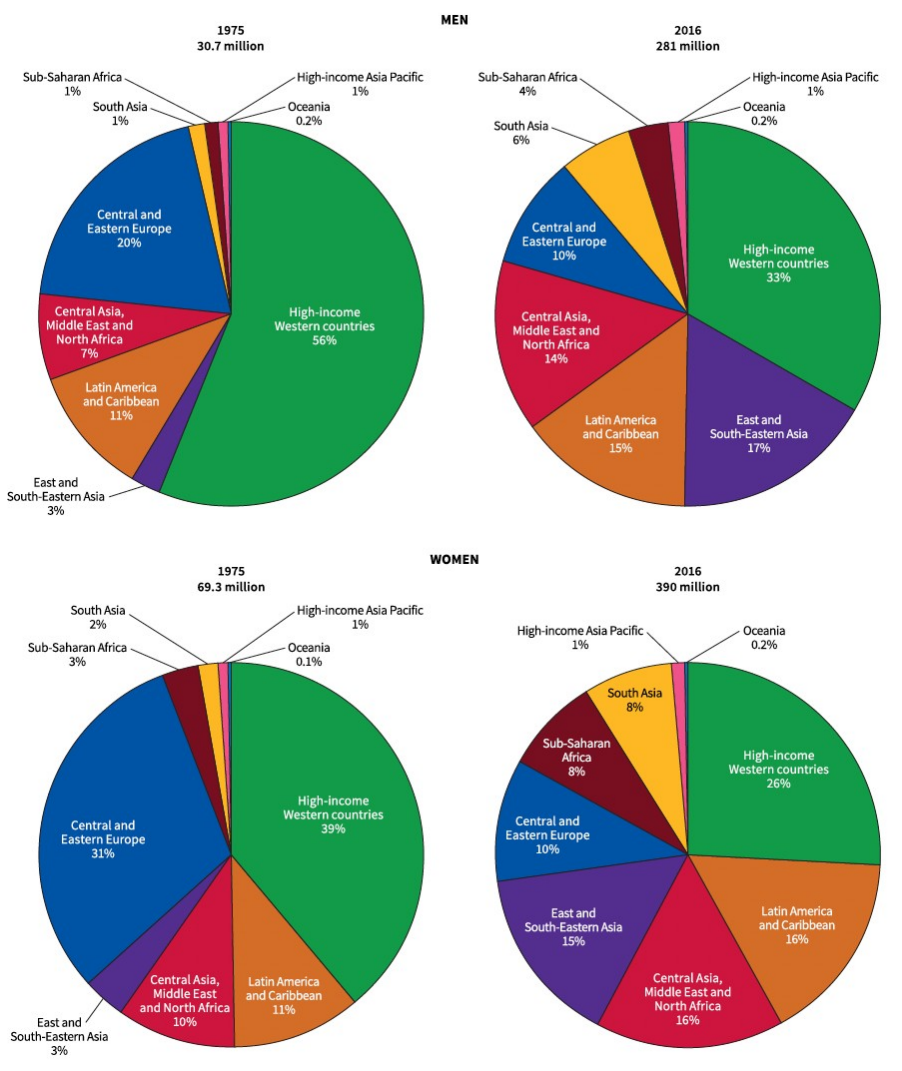
Regional contribution to global obesity burden among adults by sex in 1975 and 2016. Obesity was defined as a body mass index ≥30 kg/m^2^. Reprinted with permission from [3]. Copyright © 2023 Wiley.

Between 1975 and 2016, the largest absolute increase in the prevalence of obesity occurred in women in North Africa, the Middle East and Central Asia (from 12% to 35%), and in men in high-income Western countries (from 9% to 30%) [2,3] Figure 1. In 2016, the highest prevalence of obesity in men was reported in Polynesian Oceanic countries (range, 41–60%) followed by the United States, Canada, Australia, New Zealand, Kuwait, Qatar and Saudi Arabia (range, 31–37%). The highest prevalence of obesity in women was in Polynesian Oceanic countries (range, 52–65%), followed by South Africa, Puerto Rico, Bermuda and several Middle Eastern countries (range, 40–50%) [3]. The US Centres for Disease Control National Health and Nutrition Examination Survey (NHANES) showed that the prevalence of adult obesity in the USA increased from 30.5% in 1999–2000 to 41.9% in 2017–March 2020, with similar pre-COVID-19 obesity rates of 41.5% in adults aged >60 years, 44.3% in adults aged 40 to 59 years and 39.8% in adults aged 20 to 39 years [5]. The percentage of adults in the USA with severe or class III obesity was 6.1% for adults >60 years, 10.7% for 40–59 years and 9.7% for those aged 20–39 years of age [5].

The 2019 Global Burden of Disease systematic review reported that high BMI was related to physical inactivity, excess caloric intake, and diet quality, which is also thought to be important in cancer aetiology [4,5]. Globalization of food production and distribution, increased protein and fat availability, refined sugarcane sucrose and high-fructose corn syrup sweetening of food and beverages and decreased complex carbohydrate and dietary fibre consumption are important contributors. Such ‘Western style’ diets are typically energy dense but nutritionally deficient, with minimal wholegrain cereals, nuts, seeds, legumes, fish, fresh fruit and vegetables [1,2,3,4,5,6,7,8,9,10]. An increasingly sedentary lifestyle with declines in household, vocational and transport-related activity also coincided with the emergence of obesity in developed and developing countries [3].

Obesity-related co-morbidity includes hyperlipidaemia, type II diabetes (T2DM), atherosclerotic cardiovascular disease, non-alcoholic steatohepatitis (NASH), arterial hypertension, obstructive sleep apnoea (OSA), osteoarthritis, chronic kidney disease, neuropsychiatric conditions and premature mortality [1,2,3,4,5,6,7,8,9,10,11,12,13,14,15,16,17,18]. It is not only the global overconsumption of calories, which increased from a mean of 2390 to 2710 kcal/day between 1980–2013, but the food source that contributes to obesity and metabolic syndrome [3]. For example, the use of high-fructose corn syrup to sweeten beverages, processed food and dairy products began in the USA in the 1970′s and now comprises on average 10% of the total calories consumed per day. High-fructose corn syrup does not elevate insulin or leptin levels as glucose does, and thus stimulates hepatic de novo lipogenesis, contributing to NASH and insulin resistance [3]. The synthesis of long-chain or saturated fatty acids (palmitic acid, palmitoleic acid, stearic acid, oleic acid) from high-fructose corn syrup-sweetened beverages as part of colonocyte de novo lipogenesis and metabolic reprogramming is also associated with KRAS-mutated colorectal carcinogenesis [19].

### 3.2. Obesity-Associated Cancer

**Figure 2:**
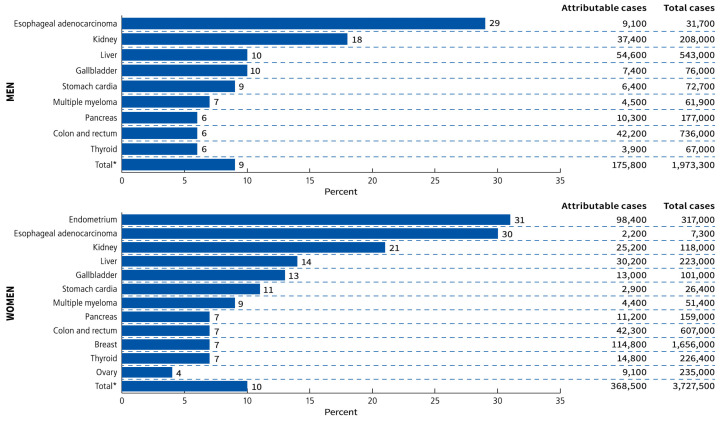
Proportions and numbers of cancer cases attributable to excess body weight (Body Mass Index ≥ 25 kg/m^2^) by sex and cancer type in 2012. * Total percentage is calculated among the excess body weight-related cancers listed in the figure rather than among all cancers. Figure reprinted with permission from [3]. Copyright © 2023 Wiley. Data source re-use with permission from Pearson-Stuttard, J.; Zhou, B.; Kontis, V.; Bentham, J.; Gunter, M.J.; Ezzati, M. Worldwide burden of cancer attributable to diabetes and high body-mass index: A comparative risk assessment. *Lancet Diabetes Endocrinol.* **2018**, *6*, e6–e15.8. Copyright © 2023 Elsevier.

In 2016, the International Agency for Research on Cancer (IARC), after reviewing more than 1000 epidemiological studies, reported that there was sufficient evidence that obesity was associated with an increased risk of 13 cancers. These obesity-associated cancers (OAC) include postmenopausal breast cancer, endometrial, ovarian, thyroid, cardial gastric, oesophageal, hepatocellular, gallbladder, pancreatic, renal and colorectal adenocarcinoma, multiple myeloma and meningioma [14]. Recent Mendelian randomization studies have suggested a causative association with obesity in six of these cancers, including renal cell cancer, oesophageal adenocarcinoma, cancers of the colon/rectum, pancreas, ovary and endometrium [6]. In a global analysis of obesity/overweight-related cancers in 2012 using population-attributable fraction (PAF) estimates, 46% of cases occurred in high-income Western countries. This assumed a 10-year lag period from 2002. There was more than twice the number of worldwide cancers attributable to excess body weight (BMI > 25 kg/m^2^) in women (368,500 cases) compared to men (175,800 cases). Excess case numbers attributable to overweight or obesity in women were driven by endometrial cancer (98,400 attributable cases, PAF = 31%), breast cancer (114,800, PAF = 7%) and colorectal cancer (42,300, PAF = 7%). In males, the tumour types that contributed the largest numbers were hepatocellular cancer (54,600 attributable cases, PAF = 10%), colorectal cancer (42,200, PAF = 6%) and kidney cancer (37,400, PAF = 18%), with oesophageal adenocarcinoma in men having the highest PAF (9100, PAF = 29%) but the lowest overall prevalence (31,700 total cases) [3] Figure 2.

### 3.3. Pathogenesis of Obesity

Obesity is considered to be a state of chronic, low-grade inflammation that is initiated by the consumption of a high-fat (energy intake from fat > 30%) or refined carbohydrate-dense diet (high fructose/sucrose), or a combination of both [17,19]. Humans have evolved to deposit larger fat stores to compensate for periods of negative energy balance, compared to other higher primates such as chimpanzees. Adult male hunter-gatherers have an average of 10–15% body fat and females 15–25%, with wild chimpanzees having an average of 5–9% body fat. This is thought to be related to the metabolic requirements of a larger brain, longer periods of offspring maturation and breastfeeding, despite relatively higher daily average walking distances (15 km male, 9 km female) in human hunter-gatherers [20]. Studies comparing human hunter-gatherer communities, subsistence farmers and Western populations have suggested that total energy expenditure may be less important than excess energy consumption in the initiation of obesity [17,18].

Genetic predisposition can contribute to obesity in people exposed to highly processed foods and sedentary lifestyles. Obesity can be characterized as polygenic or ‘common obesity’ in 95% of cases, involving multiple genes that control thermogenesis, energy homeostasis, neurohormonal signalling and leptin-insulin interactions [7]. Syndromic (e.g., Prader–Willi syndrome) and monogenic obesity are rare conditions associated with severe childhood obesity. Most of the single-gene abnormalities in monogenic obesity and hyperphagia involve hypothalamic leptin-melanocortin pathways (LEPR, POMC, AGRP, MC4R, PCSK1, SH2B1, PHIP98, MRAP2, SIM1). Because monogenic obesity often has autosomal recessive inheritance, children from consanguineous populations with homozygous carriage of allelic mutations are over-represented [7,10,11].

Founder, isolated or consanguineous populations have provided candidate genes for the study of polygenic obesity in the wider population. The MC4R gene mutation is the most common mutation found in severe childhood obesity (5%), and it also occurs in up to 0.3% of the general population. MC4R gene mutations not only increase food intake but also food preferences for high-fat foods. Since 2007, genome-wide association studies (GWAS) in adults and children have been used to study BMI as well as fat-free mass, percentage of body fat, imaging-derived adipose tissue, leptin receptor (LEPR) and circulating leptin levels [11]. A strong association was found between elevated BMI and the carrying of a missense variant in Cyclic AMP-responsive element-binding protein 3 regulatory factor (CREBRF) gene in Polynesian ethno-racial groups, which reduces energy usage and promotes fat deposition but not T2DM [3]. Variance in the macrophage apolipoprotein B48 receptor (APOB48R) gene is associated with obesity and hypercholesterolaemia. A high-fat diet increases the expression of APOB48R and lipid uptake in adipose tissue macrophages, and when the receptor is overwhelmed by triglycerides, it may contribute to macrophage foam cell formation, endothelial dysfunction and atherothrombogenesis. The FTO gene expresses the fat mass and obesity-associated protein, also known as alpha-ketoglutarate-dependent dioxygenase, which functions to increase energy intake after food deprivation but is accentuated by low physical activity. Although it is the most prevalent obesity gene variant in the European population, it has low penetrance and confers only a modest risk of common obesity in adults, which is attenuated by extra exercise [3,7,10,11].

The ‘thrifty gene’ theory which was originally proposed to explain the high rates of T2DM and obesity in indigenous groups including Pacific Islander, North American and Australian First Nations peoples was controversial. It ignored the rapid and detrimental socio-economic and cultural changes associated with European contact. Epigenetic transgenerational inheritance of obesity susceptibility has since been associated with ancestral exposure to malnutrition, a high-fat diet or environmental toxins (e.g., endocrine disruptors). This, together with epigenetic changes associated with direct exposure and modern epidemiological patterns of global obesity, support the contribution of gene expression driving phenotypic evolution—the ‘thrifty epigenome’. The influence of sociodemographic, lifestyle and clinical risk factors also explains the poor predictive value (0.4) of the GWAS-generated polygenic score (PGS*_BMI_*) in obesity susceptibility. Epigenetic alterations include DNA methylation, histone modification, altered chromatin structure and microRNA (miRNA)-mediated regulation of mRNA [3,11,12,13].

Obesity is associated with insulin resistance, hyperglycaemia, hypertriglyceridemia, hypoxia, oxidative stress, dysfunctional mitochondria, increased glycolysis, lipogenesis, adipokine/cytokine/exosome release, angiogenesis and epithelial to mesenchymal transition (EMT). There is sustained, abnormal cytoplasmic-nuclear signalling involving membrane tyrosine kinase receptors (TKR), NF-*κ*B activation and hypoxia-inducible factor-1α (HIF-1α) stabilization, as well as many epigenetic modifications in individuals with obesity [3,12,13,18].

### 3.4. Hallmarks of Cancer

**Figure 3:**
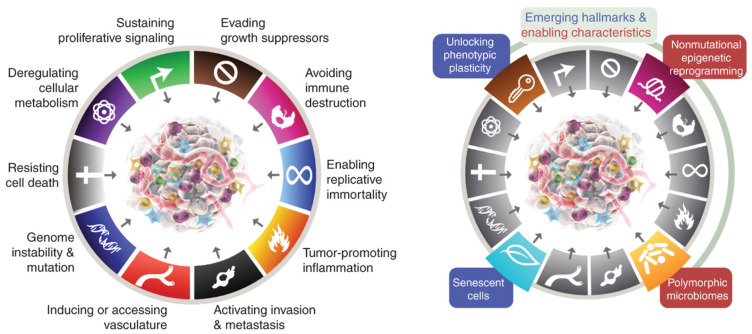
Hallmarks of cancer and New Dimensions. Ten hallmarks of cancer with 2 emerging hallmarks and 2 enabling characteristics. Obesity and carcinogenesis share many of the same molecular mechanisms. Reprinted with permission from [26]. Copyright © 2023 American Association for Cancer Research.

Such perpetual activation of inflammatory pathways and the failure of their resolution contribute to the development of cancer, as per Rudolph Virchow’s 19th century postulates [18,19,21,22,23,24,25]. The discovery of membrane tyrosine kinase receptors, the Rous sarcoma virus and the c-src oncogene after the pioneering work of Peyton Rous in 1909 showed that cancer could be an acquired disease related to the activation of oncogenic pathways by environmental stimuli, rather than solely an inherited genetic disease [25]. Otto Warburg’s original theory that cancer developed as a result of dysfunctional mitochondria and increased cytoplasmic glycolysis is also supported by the cascade of oncogenic pathways involving sustained cytoplasmic-nuclear signalling initiated by diet, obesity and the metabolic syndrome [19,24]. Harold Dvorak’s 1986 paper “Tumours: wounds that do not heal” described how cancers ‘hijacked’ the normal processes of healing, including VEGF signalling and angiogenesis during chronic inflammation [21]. The six ‘hallmarks of cancer’ were defined by Hanahan in 2000, and in 2011 four more characteristics were added, including tumour-promoting inflammation; reprogramming of cellular metabolism; avoidance of immune destruction; and genomic instability and mutation. In 2022, two emerging cancer hallmarks, phenotypic plasticity and cellular senescence, and two enabling characteristics, polymorphic microbiome and non-mutational epigenetic reprogramming, were also incorporated. Obesity and carcinogenesis share many of the same molecular mechanisms [26] (Figure 3).

### 3.5. Metabolic Syndrome and Cancer

Obesity contributes to the development of metabolic syndrome (MetS), which involves increased central adiposity [27]. This can be measured by waist circumference (European male ≥ 102 cm, European female ≥ 88 cm), waist-to-hip circumference ratio or visceral fat volume. Metabolic syndrome is diagnosed if a patient has at least three of the five following criteria: abdominal obesity, hypertension, insulin resistance/impaired glucose tolerance, elevated plasma triglyceride levels and low HDL cholesterol levels [27]. An increased risk of developing colorectal, pancreatic, bladder and post-menopausal breast cancer has been associated with metabolic syndrome or elements of metabolic dysfunction, particularly hyperinsulinaemia [27,28]. A recent 2022 analysis examined the relationship between metabolic syndrome and the risk of the thirteen IARC obesity-associated cancers [18]. It pooled the results of 63 studies conducted in the United States (15), Europe (27), Asia (18) and Canada, Israel and Australia of the cancer risk in adults (all age groups > 18 years age) without MetS versus with MetS. The effect estimates for the risk of cancer (adjusted for alcohol consumption or cigarette smoking in 68% of studies) were:

1.13 to 6.73 for breast;
1.14 to 2.61 for colorectal;
1.18 to 2.50 for gastric cardia;
1.37 to 2.20 for endometrial;
1.59 to 2.13 for pancreas;
2.13 to 5.06 for hepatocellular carcinoma [27].

### 3.6. Metabolic Syndrome and VAT

Obesity and BMI do not always correlate with the development of metabolic syndrome. Approximately 20% of the obese population have a metabolically healthy obesity (MHO) phenotype, which is associated with the accumulation of subcutaneous adipose tissue (SAT) rather than visceral adipose tissue (VAT). The MHO phenotype is more commonly found in premenopausal females who are protected from cardiovascular disease and mortality until they reach menopause. MHO is associated with small hyperplastic adipocytes rather than adipocyte hypertrophy. After menopause, females accumulate more VAT and lose lean muscle, which contributes to metabolic syndrome and obesity-related co-morbidity [27,29]. Conversely, metabolically unhealthy individuals with a normal-weight phenotype (MUNW) have a greater proportion of VAT and associated insulin resistance than individuals with MHO. There is evidence that VAT in non-obese individuals responds more quickly and intensely to insulin and glucose by releasing adiponectin, leptin and tumour necrosis factor alpha (TNF-α) than SAT [29]. Patients with Asian ancestral heritage are more prone to metabolic dysfunction such as diabetes and dyslipidaemia at lower BMI levels (>23 kg/m^2^) than other ethno-racial groups (>25 kg/m^2^), and thus may fall into this MUNW category [30]. In the 2016 EPIC study, when compared to metabolically healthy/normal-weight adults, there was a higher colorectal cancer risk among MUNW (odds ratio [OR] = 1.59, 95% CI 1.10–2.28) and metabolically unhealthy/overweight individuals (OR = 1.40, 95% CI 1.01–1.94), but not among metabolically healthy overweight individuals (OR = 0.96, 95% CI 0.65–1.42) [28].

### 3.7. Metabolic Syndrome and Colorectal Cancer

Obesity and the metabolic syndrome have also been implicated in the increasing incidence of colorectal and pancreatic cancer in younger adults under 50 years old [27]. In the USA between 1995 and 2019, the percentage of early-onset colorectal cancer (CRC) in individuals under 55 years old increased from 11% to 20% and was largely related to an increased incidence of rectal cancer (15% to 28%). Increased screening detection was not the main reason for the increase in CRC in younger adults < 50 years, as many were presenting with symptoms and signs (haematochezia, abdominal pain, iron deficiency anaemia) or with advanced disease. Although germline mutations such as Lynch syndrome are associated with early-onset CRC, the majority of patients above and below 50 years old who develop CRC have sporadic forms, with risk factors including obesity, smoking, unhealthy diet and alcohol consumption. Up to 80% of early-onset CRC are microsatellite stable tumours with a higher prevalence of p53 mutations and a lower prevalence of APC and KRAS mutations compared to sporadic CRC in patients >50 years. Age-period-cohort modelling in early-onset CRC is suggestive of early exposure to risk factors [31,32]. Combining the modifiable lifestyle factors, including cigarette smoking, excess body weight, alcohol consumption, unhealthy diet (consumption of red and processed meat; low consumption of fruits/vegetables, dietary fibre) and physical inactivity, resulted in a PAF of 54.6% for CRC in adults aged >30 years of age in the USA in 2014 [32].

When the consensus molecular subtypes (CMS) were analysed for colorectal cancers in 252 patients from the Cancer Genomic Atlas–Colon Adenocarcinoma (TCGA-COAD) database, obesity-induced enrichment of EMT- and metabolism-related hallmark genes in mesenchymal-type (CMS4) and a greater percentage of metabolic type (CMS3) colorectal cancers were found in obese compared to normal BMI patients [33]. The transcriptome-based CMS classification includes CRC with varying KRAS mutations (23% in CMS1, 28% in CMS2, 68% in CMS3 and 38% in CMS4) [34]. However, in a larger study involving pooled data from 11,872 CRC cases and 11,013 controls from 11 observational studies within the Genetics and Epidemiology of Colorectal Cancer Consortium (GECCO) and Colon Cancer Family Registry (CCFR), it was found that higher BMI increased the risk of *all* molecular subtypes of CRC (Jass type 1–4), except for Jass type 5 (Familial MSI high/Lynch syndrome) CRC [35]. The Jass classification of CRC includes:

Type 1 (‘sporadic’-MSI-H, CIMP-high, BRAF-mutated, KRAS-wild type),
Type 2 (MSS/MSI-L, CIMP-high, BRAF-mutated, KRAS-wild type);
Type 3 (MSS/MSI-L, CIMP-low or negative, BRAF-wild type, KRAS-mutated),
Type 4 (MSS/MSI-L, CIMP-low or negative, BRAF-wild type, KRAS-wild type);
Type 5 (‘Lynch syndrome’-MSI-H, CIMP-low or negative, BRAF-wild type, KRAS-wild type).

### 3.8. Dennis Burkitt, Western Diets, Obesity and CRC

Denis Burkitt and colleagues pioneered the concept of the detrimental effects of an urbanized ‘Western-type’ diet and the development of chronic diseases including T2DM, obesity, coronary artery disease, gallstones, colonic diverticulosis and colorectal cancer in industrialized countries such as Britain and the USA [36,37,38,39,40]. Burkitt highlighted the observation that between 1860 and 1960 the consumption of sugar doubled, the consumption of fat increased by 50% and the intake of dietary fibre decreased by 90%. This was related to mechanical milling of cereal grains, production of bran-deficient white bread and white rice, refining of sugar cane and corn and increased meat consumption. Burkitt advocated for the consumption of greater than 50 g/day of dietary fibre, when the average consumption in Britain was 15 g/day [36,37,38,39]. His dietary fibre in the prevention of CRC hypothesis was supported by large epidemiological studies but there was discordance with some RCTs and prospective cohort trials. Some of this was ascribed to differences in methodology, adjustments for other risk factors, separation of fibre from wholefoods and the volume and type of fibre consumed. Subsequent epidemiological work and advances in immunology and oncology have supported the concept of Western diets contributing to colorectal cancer. Dietary fibre and whole grain consumption are thought to decrease the risk of colorectal cancer by bulking stool, decreasing intestinal transit time, binding carcinogens and oestrogens and increasing their excretion. Dietary fibre promotes carbohydrate fermentation by intestinal microbiota, suppresses oncogenic signalling, colonic mucosal proliferation markers and high-risk adenomatous polyp formation. It also has metabolic benefits in decreasing cholesterol absorption and helping prevent obesity, hyperlipidaemia, atherosclerosis and T2DM [39,40,41,42,43,44,45,46,47,48].

### 3.9. Molecular Mechanisms of Obesity-Related Carcinogenesis

Most of the molecular mechanisms that induce obesity are also involved in the fourteen hallmarks and enabling characteristics of cancer, with the fundamental process being consumption of an energy-dense, highly processed diet and deposition of fat in adipocytes and hepatocytes. The metabolic and immunological responses to these triglyceride and cholesterol-laden cells, together with profound changes induced by HFD in the host intestinal microbiome, create a metabolic wound that never heals and senescent cells that never die [26] Figure 3. Failure of removal of senescent cells is a key feature of ageing, atherosclerotic cardiovascular disease, neurodegenerative diseases, obesity, chronic inflammation, NASH and carcinogenesis. Rudolf Virchow was the first to describe lipid-laden macrophages or ‘foam cells’ in atherosclerotic plaques and their contribution to inflammation. These are analogous to the macrophages that surround senescent adipocytes and hepatocytes in obesity and form crown-like structures (CLSs) in white adipose tissue (WAT) and in the liver. Lipid droplets form in macrophages when their capacity to process, transport and excrete triglycerides or cholesterol is exceeded by lipid accumulation, which results in the foamy appearance of these macrophages. This is related to acquired macrophage senescence, with impaired lipolysis and lysosomal dysfunction, as well as inadequate cholesterol trafficking, impaired lipophagy and downregulated reverse cholesterol transport [23,49].

**Figure 4:**
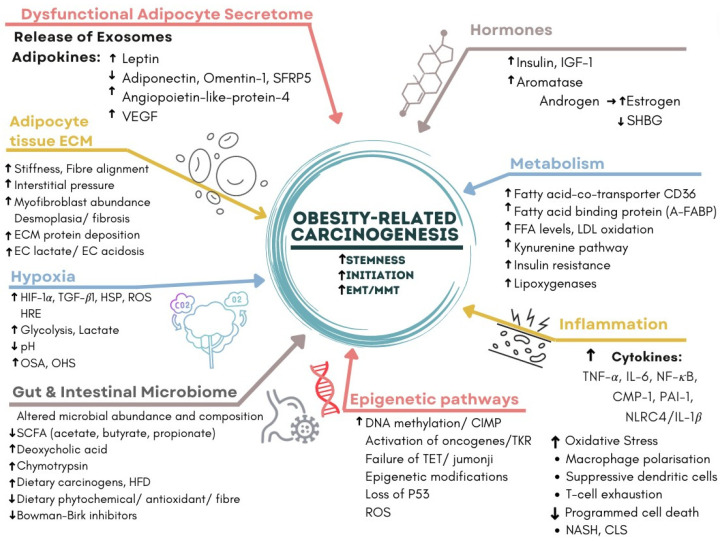
Multifactorial nature of obesity-related carcinogenesis. This involves 7 categories, which are closely related to the 14 Hallmarks of Cancer and enabling characteristics. The acronyms include: A-FABP, adipose fatty acid binding protein; CD36, Cluster of differentiation; CIMP, CpG dinucleotide island methylator phenotype; CLS, crown-like structure; CMP-1, chemoattractant monocyte protein-1; ECM, extracellular matrix; EMT, epithelial-mesenchymal transition; HFD, high-fat diet; HIF-1α, hypoxia inducible factor; HSP, heat shock protein; IGF-1, insulin-like growth factor-1; IL-1, interleukin-1; IL-6, interleukin-6; LDL, low density lipoprotein; MMT, mesothelial-mesenchymal transition; NASH, non-alcoholic steatohepatitis; NF-*κ*B, nuclear factor kappa light chain enhancer of activated B cells; NLRC4, nucleotide-binding domain and leucine-rich repeat receptor family CARD domain containing 4; OHS, obesity hypoventilation syndrome; OSA, obstructive sleep apnoea; PAI-1, Plasminogen activator inhibitor-1, ROS, reactive oxygen species; SCFA, short chain fatty acids; SFRP5, secreted frizzled-related protein 5; SHBG, sex hormone binding globulin; TGF-*β*1, transforming growth factor-*β*1; TET, ten-eleven translocation methylcytosine dioxygenase; TKR, tyrosine kinase receptor; TNF-α, tumour necrosis factor-*α*; VEGF, vascular endothelial growth factor. Adapted from [18]. Cellular mechanisms linking cancers to obesity by Liu et al. [18] is licensed under a Creative Commons Attribution 4.0 International License.

A Western-style diet, high in saturated fats and refined sugar (sugar cane, high fructose corn syrup) and low in fibre and micronutrients, contributes to obesity and obesity-associated carcinogenesis via WAT expansion, chronic inflammation and the disruption of normal homeostasis [3]. This involves the following interrelated mechanisms, described in Figure 4:

(1) Dysfunctional Adipocyte secretome: senescence, CLS, adipokines and exosomes.
(2) Hormones: insulin, oestrogen, CLS and aromatase activity.
(3) Inflammation and metabolism: CLS, cytokines and glycolysis.
(4) Hypoxia: HIF-1α, CLS, EMT, angiogenesis, VAT and OSA [50].
(5) WAT and Hepatocyte Extracellular matrix: CLS, NASH, fibrosis and cirrhosis [51].
(6) Epigenetic pathways: DNA methylation, CIMP and loss of tumour suppressors.
(7) Gut, Diet and Intestinal Microbiome: fatty acids, fermentation, fibre, bile acids and carcinogens [52] (Figure 4).

## 4. Dysfunctional Adipocyte Secretome

### 4.1. Adipocytes and Fat Storage

Obesity is characterized by excessive energy intake from Western-type diets, hyperinsulinemia, deposition of triglycerides, accumulation of WAT and hypertrophy of adipocytes in VAT and SAT [53]. In animal models and human studies of obesity and insulin resistance, VAT undergoes greater adipocyte hypertrophy than SAT, with a higher degree of macrophage infiltration, more intense levels of inflammation and loss of T regulatory cells (Treg) [54]. Brown adipose tissue (BAT) only makes up 1–2% of total body fat but is important for non-shivering thermogenesis via uncoupling protein 1 (UCP-1) on cold exposure, or *β*-adrenergic stimulation. Beigeing or browning of sub-populations of white adipocytes can also contribute to cold-induced thermogenesis via mitochondrial fat oxidation, thereby protecting against obesity [55,56,57]. The majority of dietary animal and plant fats are triglycerides, which contain saturated fatty acids (animal fat, hydrogenated vegetable oil, palm oil, coconut oil) or unsaturated fatty acids (fish, vegetable oil).

Triglyceride deposition is an efficient and anhydrous way of storing excess energy substrates compared to glycogen storage. Triglycerides circulate in the blood as apolipoproteins and undergo hydrolysis to produce free fatty acids for *β*-oxidation and glycerol. As compared to the liver and skeletal muscle, adipocytes lack glycerol kinase and thus have to synthesize glycerol-3-phosphate from glucose or fructose via cytoplasmic glycolysis or by gluconeogenesis from pyruvate in order to manufacture triglycerides. Fatty acids are obtained by adipocytes from insulin-stimulated lipoprotein lipase action on circulating VLDL or by de novo synthesis. Glycerol is combined with three esterified fatty acids to form triacylglycerides and stored as a single fat droplet in the cytoplasm of the adipocyte, squeezing the nucleus to the periphery of the cell. Insulin normally promotes lipogenesis by stimulating glucose and fatty acid uptake by adipocytes, fatty acid synthase (FAS) production of palmitate and WAT triacylglyceride deposition, and inhibits adipocyte hormone-sensitive lipase and lipolysis. Insulin counterregulatory hormones include glucagon, adrenaline, cortisol and growth hormone [58,59,60,61].

Enlargement of fat storage in adults is achieved mainly by adipocyte hypertrophy in WAT rather than adipocyte hyperplasia. Adipocyte precursor development (adipogenesis) is regulated by CCAAT enhancer binding protein alpha (C/EBP*α*), PPAR*γ* and sterol regulatory element-binding protein (SREBP)-1c. The diameter of individual human adipocytes ranges from <20 to 300 µm, and thus the stored triglyceride volume can be increased by up to a thousand-fold. Pathological adipocyte hypertrophy is associated with excessive levels of long chain FFAs, impaired *β*-oxidation of FFA and insulin resistance. Peroxisome proliferator-activated receptor-*γ* (PPAR*γ*) is inhibited and adipogenesis is suppressed during adult obesity and the loss of adiponectin. This also limits WAT storage capability or ‘fat buffering’ ability and promotes ectopic fat deposition in the liver, pancreas, heart and skeletal muscle and metabolic syndrome. It explains the beneficial effect of the PPAR*γ* agonists thiazolidinediones (pioglitazone, rosiglitazone) on insulin sensitivity in SAT but not in VAT [58,59,60,61,62].

### 4.2. WAT Senescence

Excessive WAT hypertrophy is associated with progressive ischemia, endoplasmic reticulum or cellular stress and impaired mitochondrial oxidative capacity in adipocytes. This results in their death or adoption of a senescence-associated secretory phenotype (SASP). Endoplasmic reticulum stress in adipocytes is related to exposure to circulating oxidized low-density lipoprotein (oxLDL) and dyslipidaemia [60,61,63,64,65]. Cellular senescence involves cell cycle arrest, inhibition of autophagy, avoidance of programmed cell death pathways and release of a variety of growth factors, proteases and cytokines via direct secretion or exosomes. This elicits an immune response via damage-associated molecular patterns (DAMPS), Toll-like receptors (TLR-4) and chemokines [66]. Dead, dying or dysfunctional adipocytes become surrounded by infiltrating macrophages, forming crown-like structures (CLSs) in WAT tissue. A dysfunctional WAT microenvironment is created, characterized by hypoxia, inflammation, oxidative stress and free radical damage of DNA with associated somatic mutations and epigenetic effects. In attempting to phagocytose these large, hypertrophied adipocytes, infiltrating macrophages release the stored triglycerides and free fatty acids (FFA), which can contribute to insulin resistance and hyperinsulinemia. The release of inflammatory cytokines and saturated FFA from VAT directly into the portal vein and thence to the liver is particularly important in impaired glucose tolerance and FFA lipotoxicity. This is related to accumulation of long chain FFA metabolites such as diacylglycerol and ceramide, which inhibit insulin-receptor substrate (IRS) interactions with PI3K and promote further insulin resistance [54,58,59,60,61,62]. Cellular senescence is designed to prevent the proliferation of cells with a damaged genome; however, the failure of clearance of senescent adipocytes can lead to proliferation or secondary senescence of surrounding cells via paracrine and autocrine signalling. CLSs are more often found in VAT than SAT and are closely involved in obesity-related breast carcinogenesis [67,68,69]. WAT senescence is commonly associated with ageing, but when diabetes or obesity are present it is independent of chronological age [63].

### 4.3. Prevention and Treatment of WAT Senescence

One of the main drivers of WAT senescence is increased reactive oxygen species (ROS) generation, oxidative stress and resultant telomere shortening and DNA damage. This activates the DNA damage response (DDR) and p53/p21 pathways, resulting in cell cycle arrest and a WAT SASP, including the secretion of tumour necrosis factor-*α* (TNF-α) and IL-6, and elevated *β*-galactosidase activity. DNA damage due to ROS can be reduced by antioxidants such as N-acetyl cysteine or by exercise, which can reduce WAT senescence [66,67,68,69,70,71,72]. WAT cellular senescence (measured by SA-*β*-galactosidase activity, CDKN1a, and CDKN2a), adipocyte hypertrophy, adipokine dysregulation (increased leptin secretion, decreased adiponectin), and impaired glucose tolerance and insulin sensitivity began as early as 2 weeks after initiation of a high-fat diet and was attenuated by exercise in murine models of SAT and VAT senescence. Exercise also significantly reduced the high-fat diet-induced expression of profibrotic genes in VAT, including transforming growth factor *β*1 (TGF-*β*1), fibronectin (Fn1), and tissue inhibitor of metalloproteinase 1 (TIMP1), compared to sedentary animals fed an HFD [71]. Furthermore, high-intensity interval training (HIIT) or endurance exercise (END) when administered concurrently with a high-fat diet were able to improve lean mass as a proportion of body weight (Lean mass/BW) by 14%, improve insulin sensitivity by 22% and prevent the increase in body weight (END: 17%, HIIT: 20%) and total body fat mass (END: 46%, HIIT: 50%) compared to sedentary animals in a murine model [72].

White adipose tissue SASP can be reversed by metformin treatment, which rapidly blocks the release of inflammatory adipokines and inhibits the senescent transcriptional program, leading to adipose cellular quiescence [65]. Recent clinical research has suggested senolytic agents, such as a combination of the flavonoid quercetin and the Src family tyrosine kinase inhibitor dasatinib, promote apoptosis in senescent cells by interfering with pro-survival networks including ephrin dependence receptor signalling, PI3K–AKT and BCL-2 family members. A combination of oral quercetin and dasatanib in patients with obesity, diabetes and CKD resulted in significantly reduced numbers of SAT *β*-galactosidase positive senescent adipocytes (−62%), SAT CD68+ macrophages (−28%) and SAT CLS (−86%), as well as circulating plasma SASP factors (IL-1*α*, IL-2, IL-6, IL-9, MMP-2, MMP-9, MMP-12). Because of their prolonged effect on cellular senescence, short term, intermittent senolytics (‘hit and run’ dosing) can be used, rather than continual dosing. This also helps to prevent serious adverse side effects, particularly of dasatinib and navitoclax [66].

### 4.4. Adipokines

**Figure 5:**
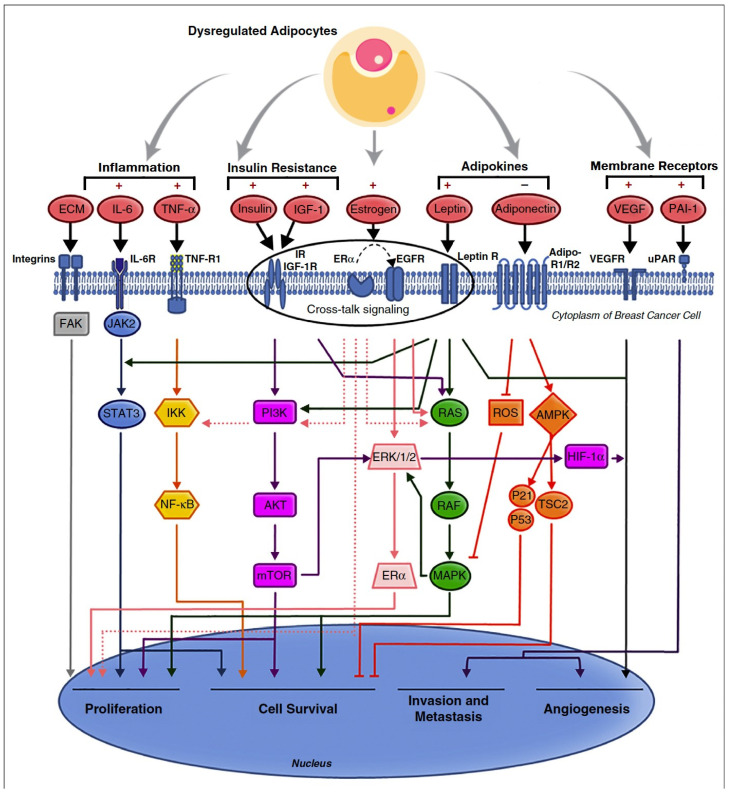
Pathways of obesity-related breast cancer due to dysfunctional adipocyte secretome, with activation of PI3K/AKT/mTOR, JAK/STAT, NF-*κ*B, RAS/RAF/MAPK and HIF/VEGF/PAI pathways. The central role of crosstalk between ER-α, IGF-1R, insulin, leptin and human EGFR in activating breast cancer cellular proliferation, survival, invasion and angiogenesis is emphasized. (Reproduced with permission from [73]). Copyright © 2023 Elsevier.

The dysfunctional adipocyte secretome is characterized by the abnormal release of adipokines, which causes detrimental effects on cellular metabolism and proliferation, energy homeostasis and insulin sensitivity. There is an increase in the release of pro-inflammatory adipokines including leptin, insulin-like growth factor-1 (IGF-1), angiopoietin-like-protein-4 (ANGPTL4), MCP-1, IL-8, IL-6, IL-1*β*, PAI-1, MIP-2 (CXCL2), TIMP and vascular endothelial growth factor (VEGF) in response to adipocyte cellular stress and hyperinsulinemia [72,73,74,75,76,77]. These adipokines affect local WAT and distant organs by promoting further adipose hypertrophy, insulin resistance, dyslipidaemia and lipogenesis and inhibiting lipolysis, fatty acid metabolism, browning/beigeing of WAT and UCP-1 expression [74] (Figure 5). Other adipokines include platelet-derived growth factor-BB (PDGF-BB), granulocyte-colony stimulating factor (G-CSF), hepatocyte growth factor (HGF), resistin, autotaxin and lysophosphatidic acid (LPA) [64,75].

**Figure 6:**
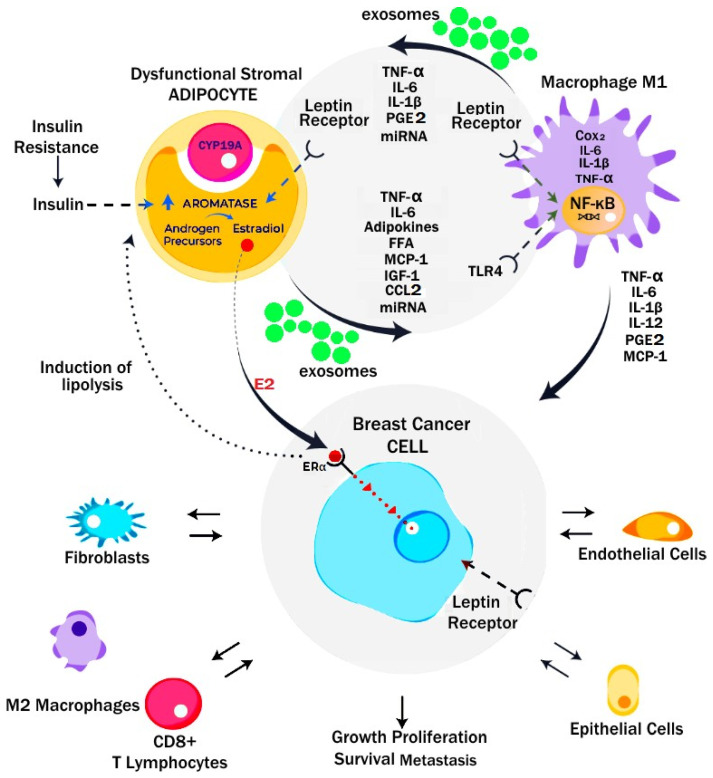
Microenvironment of dysfunctional adipocytes and breast cancer in obesity. Dysfunctional adipocytes release adipokines, cytokines and exosomes which drive a pro-inflammatory M1 phenotype in macrophages. Generation of PGE2 by tissue cyclo-oxygenase, leptin, IL-6, TNF-α and NF-*κ*B, and loss of p53 all contribute to activation of CYP19A and production of aromatase. This leads to release of 17-*β*-oestradiol and activation of ER-α receptors on ER-positive breast cancer and GPER in triple-negative breast cancer. Crosstalk between cancer cells and stromal adipocytes and macrophages promotes tumour growth, survival and proliferation. In addition, recruitment of other stromal cell elements by cancer cells enables angiogenesis, evasion of T-cell immunosurveillance and chemotherapy resistance in the tumour microenvironment. (Reprinted/adapted with permission from [73]). Copyright © 2023 Elsevier.

**Figure 7:**
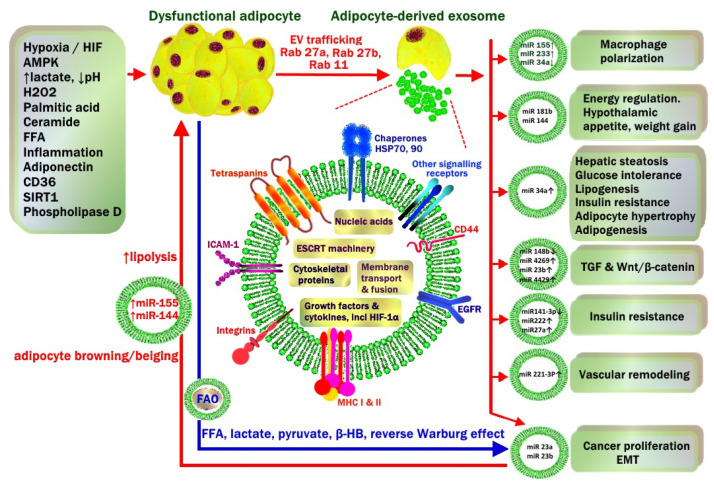
Environmental and metabolic factors that drive dysfunctional adipocyte release of exosomes in the progression of metabolic syndrome and obesity-related carcinogenesis. This is closely related to a hypoxic, acidic, inflammatory microenvironment with high levels of oxidative stress and free radical generation. Exosome cargo includes miRNA, endosomal sorting complexes required for transport (ESCRT), cytoskeleton proteins, growth factors (TGF-1*β*, TNF-α, TRAIL) and receptors (EGFR), lipids, secretory signal peptides and enzymes for lipogenesis or lipolysis. During obesity, exosomal miRNA from white adipocyte tissue affect local WAT as well as cells in distant organs, including stimulation of M1 macrophage polarization, hypothalamic energy intake, WAT lipogenesis, hepatic steatosis, insulin resistance, vascular proliferation and carcinogenesis. Crosstalk from cancer cells also involves transfer of miRNA back to cancer-associated adipocytes (CAA). This stimulates adipocyte glycolysis and lipolysis and transfer of high-energy substrates including free fatty acids (FFA), lactate, pyruvate, beta-hydroxy butyrate (*β*-HB) to cancer cells to continue mitochondrial production of ATP (Reverse Warburg effect). Fatty acid oxidase (FAO) enzymes to assist cancer cells to utilize FFA are transferred from CAA to cancer cells via exosomes. Adapted with permission from [78]. Copyright © 2023 Springer Nature.

### 4.5. Leptin

Leptin is primarily produced by SAT and is increased in individuals with higher total body fat [77]. Leptin is an anorexigenic cytokine due to its activation of pro-opiomelanocortin (POMC)-expressing anorexigenic neurons and suppression of the neuropeptide Y/agouti-related peptide (NPY/AgRP)-expressing orexigenic neurons in the arcuate nucleus of the hypothalamus. Thus, the normal effect of leptin is decreased food intake and increased energy expenditure. Under normal conditions, leptin and insulin both inhibit the effects of orexigenic stimulants (e.g., ghrelin) on the hypothalamic arcuate nucleus. Obese patients develop hypothalamic leptin resistance, reducing their sensitivity to normal satiety signals following a meal and contributing to further weight gain. Mean serum leptin levels are markedly elevated (31.3 ± 24.1 ng/mL) in obese patients compared to levels in normal-weight subjects (7.5 ± 9.3 ng/mL) [76,77]. A chronically elevated leptin level impairs post-prandial GLP-1 release and insulin sensitivity, contributing to hyperinsulinaemia in patients with normal fasting plasma glucose (pre-diabetes) and promoting the development of T2DM and NASH [79,80]. Leptin resistance can be reversed by resveratrol, metformin, GLP-1, GLP-1 analogues (semaglutide) and heat shock protein 90 (HSP-90) inhibitors [77,81].

### 4.6. Leptin and Cancer

Leptin is associated with the initiation and promotion of EMT in breast, gastric, lung, ovarian, endometrial and oesophageal adenocarcinoma, particularly in obesity [82,83,84,85,86,87]. Many of the signalling pathways that converge on EMT are activated by leptin binding to its receptor Ob-Rb. EMT is a feature of (1) embryogenesis, (2) recovery from tissue trauma or inflammation or (3) carcinogenesis. These three processes each involve stem cell differentiation or de-differentiation of mature cells. EMT is a normal process of healing or fibrosis which is hijacked by cancer cells. Cells acquire immortality due to prolonged or severe cellular stress and inflammation, related to the acquisition of mesenchymal properties, resistance to anoikis, migratory capacity and invasion of the extracellular matrix, chemoresistance, immune evasion, stemness characteristics and metabolic reprogramming, all of which are involved in cancer progression and metastastic disease [26] Figure 3.

Leptin activation of Ob-R, STAT3 and induction of Yamanaka pluripotency transcription factors OCT4 and SOX2 are thought to be involved in obesity-related tumour growth. Leptin promotes the long stabilization of HIF-1α by inhibiting SIRT-1 and p53, and leptin transcription is reciprocally promoted by the hypoxia response element (HRE) [82,83,84]. Leptin helps to suppress the stable anchoring of mature cells and allows the development of an elongated mesenchymal phenotype by promotion of EMT transcription factors Snail, Slug, Zeb1 and Twist. This results in the repression of epithelial E-cadherin and occludin and the promotion of mesenchymal N-cadherin, fibronectin and vimentin. Leptin stimulates RhoA-ROCK pathways and mixed metalloproteases (MMP), and promotes invadopodia, actin cytoskeleton reorganization and focal adhesion formation which are involved in extracellular matrix (ECM) invasion and migration [82,85].

Leptin is closely related to obesity, features of T2DM and the progression of breast cancer. Leptin drives breast cancer cell proliferation while increasing GLUT-1 mRNA levels [86]. Other T2DM-associated molecular changes include elevated insulin, interferon gamma (INF-*γ*) and oxidative stress (ROS), which contribute to further GLUT-1-mediated progression of both ER-positive and triple-negative breast cancer (TNBC) [86]. Leptin has greater effects on ER+ breast cancer proliferation and EMT than in ER− breast cancer. This is thought to be due to increased expression of the membrane leptin receptor (ObRl/Rb) in ER+ breast cancer cells and its co-localization with the ER-α, together with leptin effects on breast cancer stromal aromatase activity [87] (Figure 5 and Figure 6).

### 4.7. Anti-Inflammatory Adipokines

Conversely, there is a decrease in the levels of anti-inflammatory cytokines such as adiponectin, omentin-1 and secreted frizzled-related protein 5 (SFRP5) in obesity [88]. Adiponectin levels are negatively correlated with increasing BMI, with mean plasma levels of adiponectin in non-obese individuals (8.9 mg/mL) more than double those of obese individuals (3.7 mg/mL, *p* < 0.0001) [76,89]. Adiponectin stimulates the adiponectin membrane receptors adipoR1 and adipoR2, which increases FFA oxidation in the liver and skeletal muscles. This helps to prevent hepatic steatosis [76]. Adiponectin has anti-inflammatory properties and regulates peripheral glucose metabolism by sensitizing adipose tissue to insulin. Lowered levels of adiponectin and omentin-1 in obesity and metabolic syndrome contribute to the development of insulin resistance. The remodelling of the ECM by adipose tissue macrophages is associated with insulin resistance secondary to decreased adiponectin levels [88]. Adiponectin has antioxidant properties, which help to protect against the development of obesity, T2DM and atherogenic cardiovascular diseases [90]. Omentin-1 enhances the effect of insulin by increasing glucose uptake by VAT, activating insulin receptor substrate (IRS) proteins and inhibiting the mTOR signalling pathway. Omentin-1 also increases adiponectin gene expression and AMPK levels [88]. SFRP5 binds to Wnt proteins and blocks the action of Wnt5a, which inhibits adipose tissue-related inflammation and insulin resistance [88].

### 4.8. Adipokines and Cancer

The abnormal production and secretion of inflammatory adipokines contribute to tumour growth and progression via crosstalk signalling between adipocytes, macrophages and epithelial cells [64]. Leptin is secreted by adipocytes, fibroblasts and cancer cells, with autocrine, paracrine and endocrine effects in cancers. Figure 6. Leptin activates the Janus kinase 2 (JAK2)/STAT3 (via SH2B1), mitogen-activated protein kinase (MAPK), Nuclear factor kappa B (NF-*κ*B) and phosphoinositide 3-kinase (PI3K)/AKT/mammalian target of rapamycin (mTORC1) intracellular signalling pathways, which regulate EMT, cell proliferation, differentiation and cell apoptosis [72,90]. The aberrant activation of these pathways promotes tumour cell growth, proliferation and survival. Adiponectin has anti-tumour properties, inhibiting the JAK2/STAT3, MAPK, NF-κB, and PI3K/AKT/mTORC1 pathways while activating AMP-activated protein kinase (AMPK) and SIRT-1 [64]. Whilst adiponectin receptors adipoR1 and adipoR2 are expressed by both ER+ and ER− breast cancers, adiponectin appears to inhibit proliferation more effectively when it activates adiponectin receptors in ER− breast cancers [86]. A decrease in the circulating levels of adiponectin results in uncontrolled tumour cell proliferation and has been associated with multiple types of cancers [88,90,91,92,93] (Figure 5).

### 4.9. Adipocyte-Derived Exosomes

WAT-derived exosomes are secretory extracellular vesicles that have recently been implicated in obesity, atherosclerosis and carcinogenesis [93]. Plasma extracellular vesicles are made up of 80% microvesicles (100–1000 nm diameter), and 20% exosomes (20–100 nm diameter). Exosomes have a phospholipid bi-layer membrane similar to their parent cells, making them stable in plasma but also able to cross the blood–brain barrier or deliver their cargo via endocytosis to recipient cells in distant organs such as the liver and pancreas. Exosomes contain long non-coding RNA (lncRNA), functional mRNAs, miRNAs, small nuclear RNA (snRNA), transfer RNA (tRNA), DNA fragments, growth factors (TGF-1*β*, TNF-α, TRAIL), enzymes, lipids (25% cholesterol, 25% phosphatidylcholine, 10% sphingomyelin, 10% triglyceride, 6% ceramide), structural proteins (actin, cofilin, tubulin), endosomal sorting complexes required for transport (ESCRT) and secretory signal peptides. Exosome membranes contain tetraspanins (CD9, CD63, CD81), antigen-presenting molecules (major histocompatibility class (MHC) I and II), chaperones (HSP−70 and −90), adhesion molecules (ICAM-1, integrin-α and -*β*, CD44, P-selectin) and factor receptors/ligands (EGFR, TNFR, TfR, FasL) which are involved in recognition and endocytosis of exosomes by recipient cells, as well as some of their metabolic effects [78,94,95,96,97] (Figure 7).

### 4.10. WAT Exosomes in Lean vs. Obese Patients

**Figure 8:**
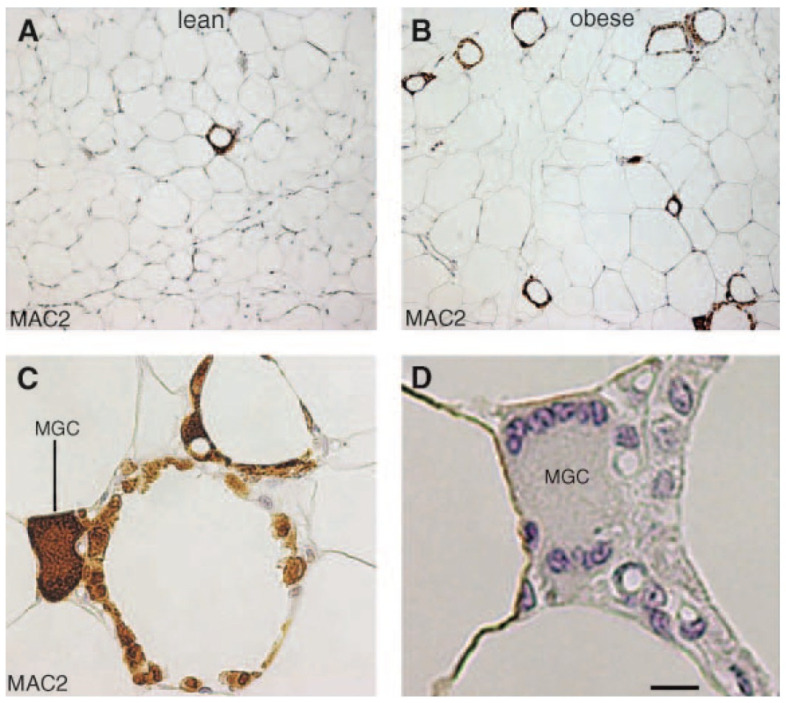
White adipose tissue (WAT) macrophages localize to crown-like structures (CLSs) around individual adipocytes, which increase in frequency with obesity. Light microscopy of visceral WAT of lean (**A**) and obese *db*/*db* (**B**) mouse showing MAC-2 immuno-reactive macrophages (brown colour) aggregated to form rare (A; lean) or numerous (B; obese) CLS among unilocular adipocytes. Note that almost all MAC-2 immunoreactive macrophages are organized to form CLS. (**C**) Enlargement of the bottom right corner of B showing that almost all mononuclear cells in CLSs are MAC-2 immunoreactive (i.e., activated macrophages). Note the multinucleate giant cell (MGC), which stains intensely for MAC-2. (**D**) Serial section consecutive to that shown in C confirming the presence of multiple nuclei (blue) in the MGC. Bar 100 mm for A, B, 28 mm for C, and 10 mm for D. Reproduced with permission from [112]. Copyright © 2023 Elsevier.

WAT exosomes in lean patients are different to those in obese patients in both cargo and number, which is related to adipocyte hypertrophy and the WAT microenvironment in obesity. Lean patients who regularly exercise produce AMPK, which suppresses exosome release from WAT by inhibiting tumour susceptibility gene 101 (TSG101) and promoting Sirtuin 1 (SIRT-1). TSG101 interacts with ESCRT machinery and scavenger receptor class B (CD36) in facilitating endosomal sorting. SIRT-1 is an NAD+-dependent protein deacetylase which normally coordinates autophagy [64]. WAT cellular stress caused by a high-saturated-fat diet (palmitate), adipocyte hypertrophy, peroxides, inflammation, hypoxia and glycolysis [98] stimulates the production of WAT exosomes (10-fold increase in number), the trafficking and release of which are controlled by the Ras-associated binding proteins (Rab) and c-Src tyrosine kinases. This is particularly associated with the lipid laden, CD9+ ATM population which accumulates in CLSs [96] (Figure 8). The protein levels of matrix metalloproteinase-2 (MMP-2), caveolae-associated protein, TGF-*β*-induced protein ig-h3, thrombospondin-1, fatty acid binding protein-4 (FABP-4), Mimecan and ceruloplasmin are elevated, and septin-11 and leptin levels are reduced, in the VAT-derived exosomes from obese subjects compared to lean subjects [76,93]. Alterations in the contents of WAT-derived exosomes such as miRNA mediate obesity via multiple pathways. For instance, adipose tissue-derived exosomes from obese patients contribute to the development of insulin resistance through the stimulation of peripheral monocytes, resulting in the release of inflammatory cytokines such as TNF-α and IL-6 [94]. Exosomes derived from dysfunctional large adipocytes have a paracrine effect on smaller recipient adipocytes to promote further lipogenesis and adipocyte hypertrophy. This is mediated by the transfer of lipogenic enzymes (acetyl-CoA carboxylase, glucose-6-phosphate dehydrogenase, fatty acid synthase) and miRNA-33/miRNA-34a in exosomal cargo. Hypoxia increases the exosome levels of these lipogenic enzymes by 3–4 times compared to normoxic conditions. Exosomes derived from obese murine and human adipocytes contain elevated levels of fatty acids, which can be transported to cancer cells together with fatty acid oxidase enzyme (FAO). These are induced by exosomal crosstalk with cancer cells, which enables fatty acid oxidation to proceed, maintaining mitochondrial activity and enhancing proliferation in cancer cells [18,64,78] (Figure 7).

### 4.11. Exosomal miRNA

During obesity, the miRNA profile of WAT-derived exosomes can be completely altered [64]. miRNA contained in exosomes released from dysfunctional adipocytes are involved in:

1. Activation of TGF-*β* and Wnt/*β*-catenin pathways (miRNA-23b, miRNA-4429).
2. Macrophage M1 polarisation (miRNA-155) and the suppression of M2 macrophage polarization (miRNA-34a).
3. Hepatic steatosis, glucose intolerance, insulin resistance (miRNA-34a).
4. Vascular remodelling and vascular smooth muscle proliferation (miRNA-221-3p).
5. Hypothalamic POMC neuronal regulation of appetite, energy intake and weight gain (miRNA-181b, miRNA-144).
6. Cancer growth/EMT (miRNA-23a) [73,78,95] (Figure 7).

The persistent release of such exosomes could not only explain the increased incidence of cancers in obese patients with metabolic syndrome, but also their poorer outcomes (more advanced cancer, shorter disease-free survival and greater risk of recurrence of cancers) than metabolically normal patients with cancer [97]. Recidivism of obesity after weight loss may also be related to permanent alterations in the WAT secretome, including exosome release [74].

## 5. Hormones

Obesity in post-menopausal women is associated with significantly higher mean levels of circulating 17-β-oestradiol (E2) compared to normal-weight post-menopausal women (21 vs. 12 pg/mL). Oestrogen production after menopause is primarily by aromatase activity in peripheral tissues (WAT, muscle, skin, endometrium). Oestrogen synthesis by aromatase in the endometrium and myometrium can be 10 times higher than in non-malignant breast tissue. In pre-menopausal women with obesity, the mean E2 levels (derived from ovarian secretion) were significantly lower than their normal-weight counterparts (32.8 vs. 39.8 pg/mL). The lower levels of E2 in pre-menopausal obesity are thought to be related to anovulatory ovarian cycles or suppression of the hypothalamic-pituitary axis with decreased gonadotrophin release. This has been proposed to explain the apparent protective effect of adult obesity on the incidence of pre-menopausal breast cancer [99].

### 5.1. Insulin and Oestrogen

Changes in hormonal expression and regulation have been associated with an increased incidence of hormone-sensitive cancers in obese women, including endometrial, ovarian and premenopausal and postmenopausal breast cancer, with a poorer prognosis and risk of recurrence after treatment [73]. For instance, insulin resistance and hyperinsulinemia secondary to obesity is associated with decreased levels of hepatic IGF Binding Proteins (IGF-BP) and elevated levels of unbound insulin-like growth factor-1 (IGF-1) [100]. The binding of IGF-1 to the intracellular insulin receptor substrate 1 (IGF-1R/IRS-1) leads to the activation of PI3K/AKT/mTORC1-network signalling and the RAS/RAF/MAPK pathways [73,99] (Figure 5). Apart from contributing to the metabolic syndrome in obese patients, this promotes tumour progression by inhibiting cell apoptosis, particularly in breast cancer. 

**Figure 9:**
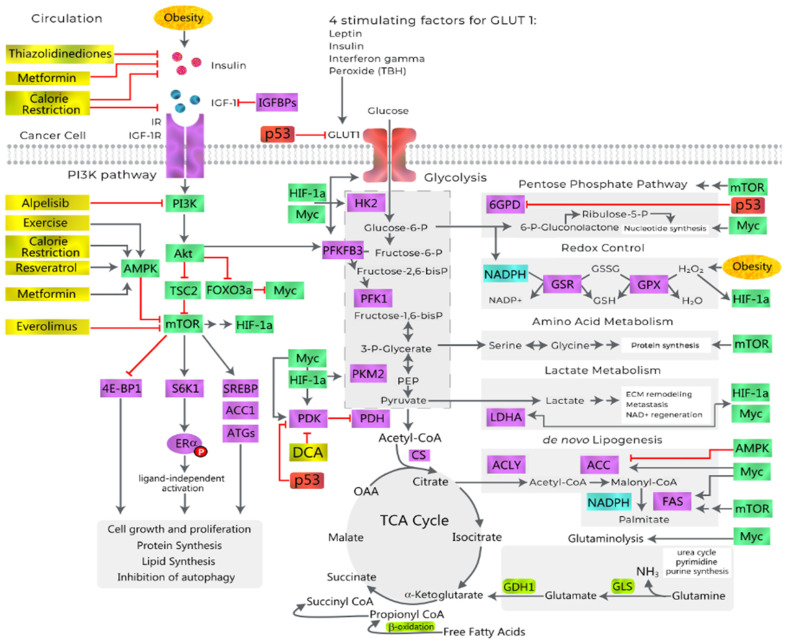
Obesity and activation of PI3K/AKT/mTORC1 pathways by insulin and IGF-1 in cancer cells. GLUT-1 is the predominant glucose transporter in cancers. Leptin, insulin, interferon-*γ* and reactive oxygen species from peroxides all stimulate GLUT-1 transport of glucose, which is fed into the hexokinase glycolysis pathway. Anabolic pathways are required for protein, nucleotide and lipid synthesis, cell growth and proliferation, and are driven by the downstream targets of mTORC1 and intracellular effects of insulin. mTORC1 also inhibits autophagy by phosphorylating proteins involved in the formation of autophagosomes (ATGs). The pentose phosphate pathway (PPP) which produces nucleotides for RNA via Ribulose-5-P, and NADPH for redox control; amino acid metabolism; lactate metabolism; and lipogenesis are controlled by AMPK, HIF-1α, mTOR and Myc. TP53 inhibits glucose flux down the PPP by regulating the dimerization of Glucose 6-phosphate dehydrogenase (G-6-PD) and inhibiting GLUT-1 and PDK. HIF-1α and c-Myc increase the activity of glucose transporters (GLUT-1), glycolytic enzymes (hexokinase-2 (HK2), phosphofructokinase (PFK1), triosephosphate isomerase (TPI1), phosphoglycerate kinase-1 (PGK-1), pyruvate kinase (PKM2) and pyruvate dehydrogenase kinase (PDHK/PDK) which inhibit PDH, driving lactate production from pyruvate via lactate dehydrogenase A (LDHA) and regeneration of NAD+. Lactate and peroxides both can stabilize HIF-1α, leading to higher activity of LDHA. Medical interventions aid in the inhibition of insulin, IGF-1 and PI3K/AKT/mTORC1/S6K1/ER-α signalling, (shown in gold). Ketone bodies, lactate and glutamine are produced by CAFs and utilized by cancer cells to maintain oxidative phosphorylation for anaplerosis. Glutamine is converted to glutamate and ammonia by GLS and then to alpha ketoglutarate by GDH-1 to enter the TCA cycle. Cytoplasmic glutamate is converted to glutathione (GSH) by glutathione synthase and used for antioxidant scavenging of ROS. Propionate is converted to succinyl-Co-A and then to succinate and fed into the TCA cycle. IGFBPs, IGF-binding proteins; IR, insulin receptor; IGF-1R, IGF-1 receptor; AMPK, AMP-activated protein kinase; TSC2, tuberous sclerosis 2; FOXO3a, forkhead box O3a; Myc, c-Myc; mTOR, mammalian target of rapamycin; HIF-1α, hypoxia-inducible factor 1-alpha; 4E-BP1, eukaryotic translation initiation factor 4E-binding protein 1; S6K1, ribosomal protein S6 kinase beta-1; SREBP, sterol regulatory element-binding protein; PFKFB3, 6-phosphofructo-2-kinase/fructose-2,6-bisphosphatase3; PEP, phosphoenolpyruvate; CS, citrate synthase; OAA, oxaloacetate; G6PD, glucose-6-phosphate dehydrogenase; GSR, glutathione reductase; GSX, glutathione peroxidase; ECM, extracellular matrix; ACLY, ATP citrate lyase; ACC, acetyl-CoA carboxylase; FAS, fatty acid synthase; DCA, dichloroacetate. (Adapted from [125]). Copyright © 2023 Doerstling, O’Flanagan and Hursting. This is an open-access article distributed under the terms of the Creative Commons Attribution License (CC BY).

The expression of insulin receptors is usually 6–10-fold higher in breast cancers than normal tissue, which when activated by insulin, also promotes cell proliferation and migration, and inhibits apoptosis via RAS/MAPK signalling [101,102,103]. The leptin receptor and ER-α are co-expressed in breast cancer tissue and engage in functional crosstalk with the IGF-1R and transactivation of EGFR. ER-α upregulates IGF pathway genes (IGF-1R, IRS, IGF ligands), and IGF reciprocally stimulates ER-α activity through serine phosphorylation of ER-α, nuclear oestrogen response element activation and target gene transcription. This is independent of E2 ligand binding and mediated by the mTORC1-S6K1 kinase axis. The PI3K/AKT/mTORC1-network can be inhibited by the PI3Kα-specific inhibitor alpelisib or by downstream blockade of mTORC1 by the rapamycin analogue everolimus. These are associated with improved progression free survival when used in combination with fulvestrant to block both ligand and non-ligand activation of ER-α in HER-2 negative, ER-positive breast cancers. Significant weight loss is an ‘on target’ effect of PI3K inhibitors, which block the catabolic intracellular action of insulin. This may be associated with an improved progression free survival (PFS), compared to patients with breast cancer who did not have treatment related weight loss (everolimus/weight loss PFS, HR 0.69, 95% CI 0.48–0.99, *p* = 0.041) [73,101,102] (Figure 5 and Figure 9). 

Insulin levels are inversely proportional to serum levels of sex hormone-binding globulin (SHBG). Thus, insulin resistance in obesity is associated with suppressed serum sex hormone-binding globulin (SHBG), which contributes to elevated serum levels of circulating free 17-*β*-oestradiol (E2) and testosterone [101]. It is estimated that the majority (70%) of the increased risk of endometrial cancer in women with obesity compared to women in the normal weight range is mediated by free E2, inflammation and hyperinsulinemia [27]. Postmenopausal patients with T2DM have a 20% higher risk of breast cancer compared to non-diabetic women, which is independent of BMI. This association is lost in premenopausal women. Elevated levels of insulin and IGF-1 also affect cancer prognosis, with worse outcomes in patients with diabetes and hyperinsulinemia [91,99,103].

### 5.2. Obesity and Aromatase

Obesity and metabolic syndrome are pro-inflammatory states characterised by the release of inflammatory mediators such as interleukin 6 (IL-6) and TNF-α. These activate the NF-*κ*B signalling pathway which results in the increased expression of the aromatase gene CYP19A1 [73]. Transcription of CYP19A1 is suppressed by p53-this is reversed by the action of both PGE2 and leptin [69,70]. Under the influence of CYP19A1, adipose tissue in obese patients has increased expression of aromatase, which upregulates the synthesis of oestrone (E1) from androstenedione and 17-*β*-oestradiol (E2) from testosterone [104]. These androgen precursors are derived from the adrenal cortex or the postmenopausal ovary. Oestrone is then converted to 17-β-oestradiol by 17B-HSD. 17-β-oestradiol activates ER-α receptors which promotes proliferation in hormone-sensitive (ER+) breast cancer cells via nuclear translocation of ER-α and the oestrogen response element. 17-β-oestradiol can also activate membrane-bound G-protein-coupled oestrogen receptors (GPER) which promotes triple-negative breast cancer (TNBC) and endometrial proliferation and cancer by activating cytoplasmic Src/EGFR/ERK signalling [69,70,91]. Local tissue levels of E2 in post-menopausal breast cancers can be 50–100 times higher than in serum due to breast WAT aromatase activity. Responses to aromatase inhibitors such as anastrozole may be inferior in obese patients with breast cancer compared to normal weight patients, and contribute to their poorer prognosis [101] Figure 6.

### 5.3. Crown-like Structures and Breast Cancer

Crown-like structures in the breast are closely related to a pro-inflammatory microenvironment and aromatization in adipose tissue and are correlated with larger adipocytes (*p* = 0.01) and higher circulating levels of C-reactive protein, leptin, insulin and triglycerides (*p* ≤ 0.05) in MUNW women [105]. Breast CLSs in 237 women who underwent mastectomy for breast cancer prophylaxis or treatment were found in 90% of obese patients (BMI ≥ 30 kg/m^2^), 53% of overweight patients (BMI 25–29.9 kg/m^2^), and 34% of normal-weight patients (BMI < 25 kg/m^2^) [106]. In a study of primary invasive HER2+ breast cancer, the presence of CD32B positive CLSs at the breast cancer-adipose tissue margin was associated with a BMI >25 kg/m^2^ and shorter time to metastatic disease in trastuzumab-treated patients (HR 4.2, 95% CI, 1.01–17.4). Trastuzumab and tamoxifen resistance are related to leptin activation of Ras/MAPK/AKT/PI3K, JAK2/STAT3, HSP-90 and overexpression of HER-2 receptors [107]. Aromatase activity in obesity is especially important in endometrial cancer, endometrioid ovarian cancer and postmenopausal ER-positive breast cancer [73,108] (Figure 6 and Figure 8).

## 6. Inflammation and Metabolism

Obesity is considered to be a state of chronic, low-grade systemic and local WAT inflammation. Hypertrophied, dysfunctional adipocytes initiate the inflammatory response, resulting in the release of inflammatory cytokines such as interleukin 6 (IL-6), tumour necrosis factor-a (TNF-a) and the activation of the nuclear factor (NF)-*κ*B signalling pathway [69]. Individuals with obesity have higher volumes of pathological VAT, with increased infiltration by macrophages attracted by adipocyte-derived monocyte chemoattractant protein (MCP-1/CCL-2) and other inflammatory chemokines [29,108,109]. The accumulation of ATM elevates the rate of glucose uptake in VAT, resulting in further inflammation, cytokine release and adipocyte dysfunction [110]. This is related to proliferating leucocytes using up to 90% of their glucose by anaerobic glycolysis, which is a more rapid but inefficient means of generating ATP by lactate fermentation compared to oxidative phosphorylation [111]. 

The majority of ATM (90%) are located around dead or dysfunctional adipocytes in WAT CLS, with CLS being observed rarely in lean wild-type mice (0.34 ± 0.28 CLS per 100 adipocytes) (Figure 8) but increased by 30-fold (10.50 ± 1.05 CLS per 100 adipocytes) in obese, leptin receptor-deficient *db*/*db* mice (*p* < 0.001). These ATM stain for MAC-2 (galectin-3), which is a 26 kDa *β*-galactoside-binding lectin recognized as a monocyte chemokine, a scavenger receptor and a mediator of local and systemic inflammation. The same 30-fold increase in CLSs was found in biopsies from SAT and VAT (omental) in obese human subjects (BMI > 30 kg/m^2^) compared to those from lean (BMI = 20–24.9 kg/m^2^) patients. A doubling of adipocyte size was correlated with adipocyte death (0.37 ± 0.08 g/lipid per cell; *n* = 15) vs. no dead adipocytes (0.17 ± 0.09 g/lipid per cell; *n* =13) (*p* < 0.001). Adipocyte death in WAT was also significantly correlated with obesity (BMI= 30–45 kg/m^2^) (*p* = 0.04), with features of necrosis rather than apoptosis [112] Figure 8.

### 6.1. Adipose Tissue Macrophage Polarization

Dysfunctional adipocytes particularly in VAT engage in crosstalk via cytokines and exosome exchange with adipocyte tissue macrophages (ATM), which results in M1-like polarisation. This is the pro-inflammatory phenotype of macrophage activation, with overexpression of macrophage-derived TNF-α, IL-1*β*, IL-6, PGE2 and inducible nitric oxide synthase (iNOS) [29]. In contrast, the VAT of lean individuals is characterised by the M2 ATM anti-inflammatory phenotype, and adiponectin release, regulatory T cells and expression of IL-10 and IL-33. IL-10 is an anti-inflammatory cytokine associated with normal insulin sensitivity and stimulation of lipid catabolism [29,109]. IL-33 binds to IL-33 receptors on Treg cells and, together with PPAR-*γ*, promotes a Treg signature and immune and metabolic homeostasis. A Treg signature in lean SAT promotes non-shivering thermogenesis via increased UCP-1 and beigeing of WAT [113].

Obesity results in the phosphorylation of PPAR-*γ* and the disappearance of Tregs and the suppression of adipogenesis in VAT [113]. There is a transition from the M2 to the M1-like macrophage phenotype in the ATM of obese individuals, which is mediated by adipocyte exosomes, NF-*κ*B, cyclooxygenase (COX)-2, Toll-like receptor (TLR) and Notch signalling pathways [73,109]. This change in phenotype has also been implicated in tumour initiation and progression. (Figure 6). Polarization of macrophages is related to M2 macrophages mainly using oxidative phosphorylation for ATP production, and M1 macrophages preferentially using cytoplasmic glycolysis and lactate formation. Indeed, inhibition of glycolysis by 2-deoxyglucose in ATM almost completely inhibited the release of ATM cytokines (IL-6) and chemokines (CXCL-1) in WAT from obese animals. Inhibition of the glycolytic enzyme phosphoglycerate kinase-1 (PGK-1) also inhibited IL-1*β* and IL-6 but not TNF-α production in macrophages activated by microbial lipopolysaccharides (LPS). Inhibition of NF-*κ*B signalling prevented M1 polarization and insulin resistance in obese *db*/*db* mice, an established model of obesity-associated WAT inflammation [112].

### 6.2. Metabolically Activated Macrophages and Crown-like Structures

However, some differences in metabolic pathways have been identified between classically activated (LPS) peritoneal M1 macrophages and metabolically activated macrophages (MMe) in obese ATM. This is possibly related to phagocytosis of dead adipocytes or autophagy providing alternative fuel sources for ATM including saturated fatty acids and glutamine. Metabolically activated macrophages are the source of the senescent, lipid laden, multinucleated, tetraspanin CD9+ macrophages found in CLSs which actively secrete exosomes and inflammatory cytokines/chemokines [23,96] (Figure 8). ATM exposure to saturated fatty acids (e.g., palmitate) increases the MMe polarization and release of IL-1β, IL-6 and MCP-1, whereas unsaturated fatty acids promote an anti-inflammatory M2 phenotype. Adipose tissue macrophages may be primed by palmitate to release increased amounts of TNF-α and IL-6 after microbial LPS exposure and TLR-4 activation. This is thought to be a type of innate immune memory which may explain the phenomenon of *weight cycling* and the risk of weight regain after weight loss in obese patients [114,115,116,117].

## 7. Hypoxia

Hypoxia can result from impaired oxygenation of haemoglobin, inadequate tissue capillary density or disrupted diffusion of oxygen to cells. Adaptive responses to hypoxia include increasing the level of haemoglobin by erythropoiesis, the number and density of blood vessels by angiogenesis or altering cellular metabolism and the utilization of energy substrates. These are mediated by both HIF-dependent and HIF-independent pathways, which also coordinate EMT programs in tissues. Late stages of WAT hypertrophy are characterized by dysregulated angiogenesis, TGF-*β* release, impaired adipocyte lipolysis in response to catecholamines and WAT hypoxia [98,118,119,120]. Crown-like structures are associated with expanding lipid storage in WAT, and contain hypoxic, glycolytic M1-like macrophages derived from peripheral blood monocytes [104,120]. CLS macrophages are associated with the release of inflammatory cytokines and FFA, which contribute to insulin resistance and hyperinsulinaemia. In murine models of Western diet-induced obesity, it was found that HIF-1α expression, stabilization and nuclear translocation occurred in CLS-associated macrophages in both SAT (mammary glands) and VAT (mesenteric fat). However, macrophages outside CLSs in murine WAT were not found to be hypoxic. Specific agonism of ER-*β* expressed on CLS macrophages was shown to suppress CLS numbers and inhibit macrophage HIF-1α activation and IL-1*β*, TNF-α and osteopontin release within CLSs. This demonstrated the anti-inflammatory and anti-proliferative effect of ER-*β* in mammary gland adipose tissue and VAT [104].

### 7.1. Hypoxia and Obstructive Sleep Apnoea

Obese individuals often have comorbid obesity hypoventilation syndrome (OHS), which is characterised by daytime hypercapnia, intermittent nocturnal hypoxia and sleep-disordered breathing including severe or moderate OSA [50]. Up to 90% of OHS patients will have OSA, and 73% have severe OSA. Around 10% of patients with OHS will have non-obstructive hypoventilation [50]. OSA has been correlated with a greater volume of VAT, level of inflammation and rate of metabolic activity in VAT, based on hybrid 18F-FDG PET/MRI imaging. Metabolic activity in VAT in patients with OSA was shown to be independent of age and BMI, which suggests that OSA is an independent risk factor for the progression of metabolic syndrome. This creates a perpetual cycle of increasing visceral fat volume, decreased insulin sensitivity and progressive OHS due to nocturnal airway obstruction, decreased respiratory system compliance, inadequate alveolar ventilation, chronic intermittent hypoxia, ROS production and HIF-1α activation. The generation of ROS is increased due to the repeated de-oxygenation and re-oxygenation cycles and reperfusion injuries sustained during chronic intermittent hypoxia and nocturnal OSA. This is associated with lipid peroxidation, oxidized LDL, depletion of antioxidant defence systems and activation of the NLRP3 inflammasome. OSA causes sleep fragmentation and increased activity of the sympathetic nervous system with associated catecholamine and cortisol release. This also contributes to insulin resistance, hyperlipidaemia and NASH [18,29,98,121]. Bariatric metabolic surgery results in substantial and sustained weight loss, reducing the severity of OSA (18–44% reduction of the AHI (apnoea-hypopnoea index)), and improvement in gas exchange (17–20% reduction in PaCO2), pulmonary hypertension and daytime somnolence, leading to the resolution of OHS and NASH [29,98,121].

### 7.2. The Warburg Effect

Hypoxia alters cell metabolism, resulting in the shift from oxidative phosphorylation to anaerobic glycolysis to generate ATP. Decreasing the activity of mitochondria in the presence of hypoxia prevents electron leakage onto molecular oxygen, the generation of superoxide and hydrogen peroxide and mitochondrial-mediated apoptosis [29,122] (Figure 9). Cytoplasmic glycolysis and the production of lactate from pyruvate occur in some rapidly proliferating cancer cells even in the presence of oxygen, and is termed the Warburg effect or aerobic glycolysis [123,124]. Enhanced glycolysis increases the production of lactate which binds to the Fe(II)/2-oxoglutarate-dependent dioxygenase enzyme prolyl hydroxylase domain containing 2 (PHD2) and inhibits its activity, preventing the hydroxylation of HIF-1α. Under conditions of normal cellular oxygen, ferrous iron (Fe^2+^), vitamin C and mitochondrial succinate, HIF-1α is hydroxylated by PHD2. It is then ubiquitinated by the von Hippel-Lindau protein (pVHL)-elonginB-elonginC (VBC) complex and subsequently degraded by the 26S proteasome. Thus HIF-1α and PHD2 normally act as homeostasis sensors for cellular oxidative stress (GSH depletion/ROS), hypoxia, redox potential (ascorbate), iron deficiency and mitochondrial dysfunction (succinate) [19,122]. HIF-1α is also degraded under normoxic conditions when it binds to the tumour-suppressor gene p53, resulting in HIF-1α ubiquitination and proteasomal degradation by mouse double minute 2 homolog (Mdm2). This process is inhibited in hypoxic tumours due to the loss of function or mutation of tumour suppressor genes such as p53, contributing to elevated HIF-1α levels [123].

### 7.3. Oscillatory HIF-1α and the Hypoxia Response Element (HRE)

Under hypoxic conditions, the inhibition of PHD2 and the stabilisation of HIF-1α result in its translocation to the cell nucleus, dimerization with HIF-1*β* and binding of the heterodimeric HIF-1 complex to the hypoxia-response element (HRE) of the gene promoter for transactivation. Excess extracellular lactate can modulate oscillations in HIF-1α via a quorum-sensing signal, protecting tumour cells from hypoxia-induced cell-cycle arrest of division, which contributes to a more aggressive pattern of proliferation [124]. Oscillatory HIF-1α activity results in the transcription of multiple hypoxia-responsive genes in the HRE, which are involved in cell metabolism, erythropoiesis, angiogenesis, cell proliferation and metastasis. This may also explain the differing effects of chronic continuous hypoxia and the chronic intermittent hypoxia which occurs in OSA. The following downstream pathways are activated by the HRE:

○ Glucose transporters (GLUT-1 and -3);
○ Glycolytic enzymes (HK-1, HK-2, PFKFB3, PGK-1, PKM-2, GAPDH, LDHA);
○ Metabolic reprogramming of mitochondria (PDHK);
○ Erythropoeisis (EPO);
○ Angiogenesis (leptin; nitric oxide synthase (NOS); vascular endothelial growth factor (VEGF); LDL-receptor-related protein 1 (LRP1); ADM, adrenomedullin (ADM); transforming growth factor-*β*3 (TGF-*β*3));
○ Chromatin methylation (TET2, jumonji family histone demethylases JMJD1A and JMJD2B);
○ Cellular detachment, EMT and migration (E-cadherin repression, Hedgehog, TGF *β*/SMAD3, ILK, Wnt/b-catenin, dynamic actin reorganization, EMT transcription factors);
○ Cell survival and resistance to anoikis (ADM, EPO, NOS-2, insulin-like growth factor (IGF-2), IGF-factor-binding protein 2 (IGF-BP2), transforming growth factor *α* (TGF-*α*));
○ Cell proliferation (IGF-2, NOS, myelocytomatosis virus oncogene cellular homolog (C-Myc); DNA-binding protein inhibitor (ID2)) [122,124].

### 7.4. HIF and Metabolism

In vitro studies have demonstrated that HIF-1α directly mediates insulin resistance in adipocytes [121]. The activation of HIF due to continuous hypoxia in adipocytes resulted in increased expression and levels of GLUT-1, with increased glycolysis and release of lactate, but decreased expression of the insulin-dependent glucose transporter GLUT-4 [29]. Many of the glycolytic enzymes (HK-2, PKM-2, PGK-1, LDHA), which are promoted by HIF-1α, also have non-glycolytic or ’moonlighting functions’ as transcription factors or protein kinases when translocated to the nucleus or mitochondria. They thereby modulate such processes as autophagy, apoptosis, resistance to anoikis, cellular attachment, cell cytoskeleton and cell cycle control. This contributes to uncontrolled cellular proliferation, a hallmark of carcinogenesis [26,126,127,128] (Figure 3, Figure 5 and Figure 9).

HIF-1α drives tumour angiogenesis by increasing the expression of vascular endothelial growth factor (VEGF) and angiopoietin-like 4. Tumour stromal cells secrete VEGF which binds to the VEGF receptor 2 (VEGFR2). This activates the PLC*γ*-PKC, MAPK-ERK1/2, and PI3K-AKT pathways resulting in cancer cell proliferation and survival [129].

Fatty acid synthesis and lipid metabolism are modified since the TCA cycle is inhibited under hypoxia. Hypoxia prevents the conversion of glucose into citrate, which is the source of fatty acid precursors and acetyl-CoA, and suppresses the activity of acyl-CoA dehydrogenases [123]. These metabolic changes have been proposed to further impair glucose tolerance, which has been associated with cancer progression [121]. Increased exposure of ATM to the saturated fatty acid palmitate during high-fat diet-induced obesity further promotes glycolysis, IL-1*β* release and HIF-1α nuclear translocation under both WAT hypoxia and normoxia [130].

### 7.5. Reverse Warburg Effect

Reactive oxygen species are released under continuous hypoxia, resulting in oxidative stress through mitochondrial dysfunction, nitric oxide synthase (NOS) uncoupling and the activation of NADPH oxidase (NOX) and xanthine oxidase (XOX) [131]. Cancer cells induce oxidative stress in cancer-associated fibroblasts (CAF) and cancer-associated adipocytes (CAA) through the secretion of cytokines and ROS (e.g., peroxides). This drives CAF/CAA to utilize glycolysis instead of oxidative phosphorylation even during normoxia, producing high-energy metabolites such as lactate and pyruvate to be utilized by cancer cells for ATP production and anabolic pathways. The metabolic coupling between cancer cells and host-derived stromal cells is known as the Reverse Warburg effect, another model that has been proposed to explain aerobic glycolysis and lactate production in cancer tissue [122].

HIF-1α is stabilised by hypoxia, ROS, PKM-2, HSP, TGF-*β*1, TNF-α, leptin or p53 mutation. Stabilised HIF-1α not only upregulates the expression of pyruvate dehydrogenase kinase (PDK/PDHK1), but also its phosphorylation and activation by enabling mitochondrial translocation of PGK-1. By acting as a protein kinase, mitochondrial PGK-1 activates PDHK1, which then inhibits PDH and the conversion of pyruvate to acetyl-CoA. This prevents the normal cycling of the tricarboxylic acid (TCA) cycle and generation of ROS in the mitochondrial respiratory chain. [125,132] Figure 9.

**Figure 10:**
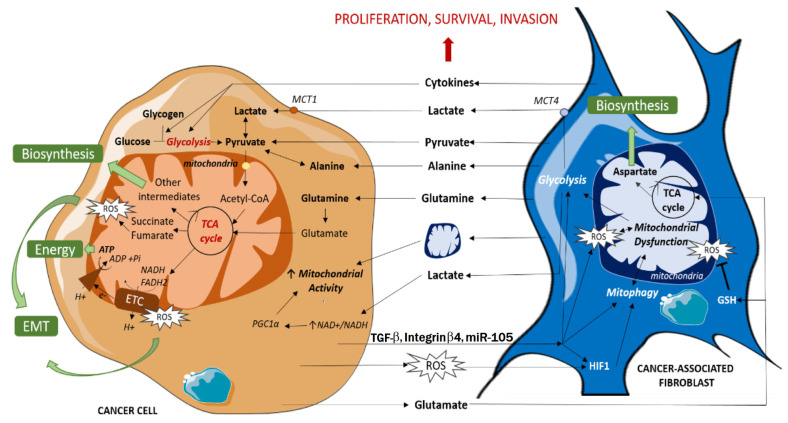
Metabolic crosstalk between cancer cells and CAF/CAAs: the reverse Warburg Effect. CAF and CAAs fuel the cancer cell TCA cycle by directly providing cancer cells with organic acids (lactate, pyruvate) and amino acids (alanine, glutamine) via increased glycolysis and monocarboxylate/amino acid transporters, or indirectly by enhancing glycolysis via cytokine release. This results in an increase in mitochondrial activity, leading to energy and biosynthetic precursor production and redox state modulation. CAFs also enhance mitochondrial activity in cancer cells via direct transfer of intact mitochondria. In turn, cancer cells induce aerobic glycolysis and mitochondrial dysfunction, mitophagy and ROS production in CAFs by TGF-*β*, integrin-*β*4 and exosomal miR-105 transfer, amplifying their mutual support. EMT: Epithelial Mesenchymal Transition, ETC: Electron Transport Chain, GSH: reduced glutathione, mtDNA: Mitochondrial DNA, MCT: MonoCarboxylate Transporter, ROS: Reactive Oxygen Species, TCA cycle: TriCarboxylic Acid cycle. Reproduced with permission from [133]. Attribution 4.0 International (CC BY 4.0).

Pyruvate, as the end product of the hexokinase pathway, is thus diverted away from mitochondria to be fermented in the cytoplasm to lactate by LDH-A and the co-enzyme NADH. Lactate fermentation produces NAD+, which is required by glyceraldehyde-3-phosphate dehydrogenase (GAPDH) to maintain the flux of glucose through the hexokinase glycolytic pathway. Lactate fermentation is important when hyperglycaemia or hyperinsulinemia overwhelm the oxidative capacity of the mitochondria, which can otherwise lead to a disrupted NADH/NAD+ ratio and mitochondrial oxidative stress [68,122,133] (Figure 3, Figure 6, Figure 9 and Figure 10).

### 7.6. Metabolic Reprogramming and Cancer

ROS release results in the inhibition of PHD2 and the upregulation of redox-sensitive transcription factors including NF-*κ*B and HIF-1α. The activation of NF-*κ*B increases cytokine release and the metabolic reprogramming of CAF/CAA, related to loss of the PHD regulation of I*κ*B kinase [131,133]. HIF-1α furthers oxidative stress in CAF/CAA via autophagy and lysosomal degradation. This results in the stromal loss of the structural protein Caveolin-1 (Cav-1), which increases ROS production via a positive feedback mechanism. The loss of Cav-1 additionally results in uninhibited nitric oxide (NO) synthesis, which causes mitochondrial dysfunction and promotes glycolysis in CAA/CAF. HIF-1α promotes the upregulation of glycolytic enzymes and mono-carboxylate transporters (MCT-4) in CAA/CAFs, thereby increasing the production and transportation of mono-carboxylates (lactate, pyruvate, beta-hydroxy butyrate (*β*-HB)) into the tumour microenvironment. These are then taken up MCT-1 in cancer cells and used in the citric acid cycle or for biosynthetic pathways (lactate/pyruvate) including gluconeogenesis, lipogenesis and ribose-5-phosphate in the non-oxidative branch of the pentose phosphate pathway (PPP). Ribose-5-phosphate is required for DNA, RNA and co-factor NAD, NADP and FAD synthesis, essential for ATP production, redox potential, and growth and cellular proliferation [133]. Transformed stromal cells (CAA/CAFs) also provide glutamine, which is then transported into cancer cells via the tumour microenvironment by sodium-linked membrane glutamine transporters (SLC1A5, SLC38A1, and SLC38A2). Glutamine is converted to glutamate by the glutaminase enzyme (GLS) with the release of ammonia. Ammonia is then used for pyrimidine, purine and amino sugar synthesis by cancer cells. The activity of GLS is enhanced by c-Myc. Cytoplasmic glutamate is converted to glutathione (GSH) by glutathione synthase and distributed in the cell for the antioxidant scavenging of ROS. Glutamate can also be converted to alpha-ketoglutarate by mitochondrial glutamate dehydrogenase 1 (GDH-1), and thus enter the TCA cycle. Some cancers rely almost entirely on glutamine anaplerosis for the generation of oxaloacetate and acetyl-CoA [133,134,135] (Figure 9 and Figure 10).

### 7.7. Regulation of Cellular Anabolism vs. Catabolism

HIF-1α is a key promoter of obesity-associated cancer due to its effect on multiple pathways that contribute to tumorigenesis. However, there are other pathways independent of HIF-1α that enable cancer cell proliferation and survival under hypoxic conditions. This includes signalling pathways involving AMPK, HIF-2, Myc, PI3K-Akt and 2-hydroxyglutarate as well as epigenetic mechanisms, post-translational protein alterations and enzyme spatial reorganization [123]. ROS affect the activity of AMPK, a heterotrimeric serine/threonine kinase, which mediates the transition from anabolic metabolism to catabolism. AMPK is activated following a reduction in the cellular AMP/ATP ratio, resulting in the production of ATP from both oxidative phosphorylation and aerobic glycolysis [136]. Both AMPK and HIF-1α are key determinates of tumour growth and progression, in the context of oxidative stress and nutrient depletion [137] (Figure 5 and Figure 9).

ROS also promote the activity of c-Src, which is involved in APC-related colorectal carcinogenesis. The flux of glucose through glycolysis is coordinated by c-Src and p53. The c-Src protein kinase phosphorylates 6-phosphofructo-2-kinase/fructose-2, 6- bisphosphatase (PFKFB3) to produce more F-2,6-BP, which then activates PFK1 and directs more G6P into glycolysis rather than the oxidative PPP. Increased glycolysis produces extra 3-phosphoglycerate (3PG), a precursor for serine and glycine synthesis, which enables tumour biosynthesis and proliferation to proceed. C-Src induced glycolysis also increases lactate production and extracellular acidification. The activation of c-Src has a feed-forward effect on ROS generation, due to an increased flux of glucose into the TCA cycle and decreased oxidative PPP generation of NADPH [138] (Figure 9).

Wild-type p53 suppresses glycolysis by decreasing the transcription of glucose transporters (GLUT-1, GLUT-4) and increasing the expression of TP53-induced glycolysis and apoptosis regulator (TIGAR), which reduces F-2,6-BP levels and limits the activity of PFK-1. Wild-type p53 also promotes the activity of PDH by inhibiting PDK1. This favours the generation of acetyl Co-A and mitochondrial oxidative phosphorylation, rather than cytoplasmic lactate production. WTp53 limits glucose flux down the PPP by regulating the dimerization of glucose 6-phosphate dehydrogenase (G-6-PD). Loss of p53 function by gene mutation or DNA methylation promotes the effects of HIF-1α and c-Src [111] Figure 9.

## 8. WAT and Hepatocyte Extracellular Matrix

Expanding adipose tissue results in the remodelling of the ECM, with increased tissue stiffness, interstitial pressure, fibre realignment and abundance of myofibroblasts [18]. Cancer-associated adipocytes display a fibroblast-like phenotype, promoting the secretion of fibronectin and collagen and restructuring the internal actin and vinculin cytoskeleton [139]. The deposition of fibronectin and collagen underpins cancer tissue desmoplasia, with the formation of a hypoxic, fibrotic tumour stroma. Transforming growth factor (TGF-*β*1), a pro-fibrotic cytokine is also activated by lactate, ROS and MMPs. This contributes to further matrix protein deposition, fibrosis and inflammation of adipose tissue, while inducing the differentiation of myofibroblasts that drive tumorigenesis. Activation and release of TGF-*β*1 can induce NADPH oxidase 4 (NOX 4) expression which causes further ROS release—a positive feedback loop called ‘redox-fibrosis’ [19,129].

### CLS and NASH

The remodelling of the ECM in obesity plays a key role in the pathogenesis of breast cancer, pancreatic ductal adenocarcinoma and NASH, hepatic fibrosis and hepatocellular carcinoma [140,141]. High-energy diets, obesity and increased triglyceride and cholesterol deposition in hepatocytes result in hepatocyte ballooning and oxidative stress, insulin resistance, ROS release, FFA oxidation and FFA lipotoxicity. Continued hepatocyte injury, SASP, autophagy and apoptosis lead to the recruitment of Kupffer cells, bone marrow-derived macrophages and hepatic stellate cells (HSC), hepatic inflammation and CLS formation, activation of IL-6/leptin/JAK2/STAT3 pathways and pericellular fibrosis. Hepatic CLS, with apoptotic hepatocytes containing cholesterol crystals surrounded by recruited macrophages, distinguishes NASH from simple hepatic steatosis. The number of hepatic CLSs also positively correlates with activated fibroblasts, collagen deposition and the degree of NASH-associated hepatic fibrosis [142].

Activated HSCs secrete leptin and also express Ob-R, which creates an autocrine and paracrine effect on HSC trans-differentiation into myofibroblasts and release of TGF-*β*1, angiopoietin-1, VEGF and collagen-I, hepatic fibrosis and angiogenesis. Kupffer cells also have membrane bound Ob-R, which when stimulated by leptin secrete TGF-*β*1. Although NASH, progressive hepatic fibrosis, ECM deposition and cirrhosis lead to the development of HCC, some patients with NASH still develop HCC without advanced fibrosis or cirrhosis. This may be due to an IL-6/STAT3 autocrine loop driving the transformation of HCC progenitor cells [56,76,140,143] (Figure 5). Hepatic susceptibility to microbial endotoxins (LPS) from the portal vein may be increased by leptin in obese individuals, contributing to the progression of NASH [144]. A threshold of >10% of total body weight (TBW) loss is important in the regression of NASH and liver fibrosis in 45% of patients, potentially preventing obesity-related cirrhosis, hepatocyte dysplasia and HCC development [51,143].

## 9. Epigenetic Pathways

Obesity results in epigenetic alterations such as DNA methylation, which has been correlated with the future development of CRC and breast cancer [145,146,147,148,149]. The other main types of epigenetic changes include histone modifications and non-coding mRNA interference. Histone modifications regulate gene expression through the addition or removal of a chemical group (acetylation, methylation, phosphorylation, ubiquitination) which affects the compaction of chromatin [149]. As discussed in the section on dysfunctional adipocyte exosomes, microRNAs control gene expression by cleaving mRNA or interfering with the production of proteins by coding RNA [145]. The epigenome of mothers with pre-pregnancy obesity can be inherited by their offspring, with evidence of increased DNA methylation influencing offspring BMI z-score and body growth, which may also be sex-specific. The obesity epigenome and transgenerational inheritance were recently reviewed by Wu et al. (2022) [148].

### 9.1. Differentially Methylated Regions

DNA methylation is the attachment of a methyl group to a cytosine residue in a CpG dinucleotide (CpG) at the gene’s promoter region. Differentially methylated regions (DMR) can involve either CpG hypomethylation leading to increased expression of a gene (e.g., oncogenes), or CpG hypermethylation, which may decrease gene expression (e.g., tumour-suppressor genes). DMR can affect the expression of genes involved in DNA repair, cell cycle regulation, cell proliferation and apoptosis, extracellular matrix, Wnt signalling, hormonal signalling and metabolism. DNA hypermethylation increases the risk of abnormal cell function and growth, predisposing individuals to develop chronic diseases, obesity and cancer [146]. Aberrant DNA methylation can result from the consumption of a high-saturated-fat diet and obesity and be reduced by dietary intervention and physical exercise [149]. Examples of DMRs in CRC include TGF-*β*2, KRAS, adenomatous polyposis coli protein 2 (APC2) and SMAD family member 3 (SMAD3). The CpG island (CGI) methylator phenotype (CIMP) can be used as a cancer biomarker for cancers with hypermethylation in CGI promoters, such as CMS1-type CRC, uterine corpus endometrial carcinoma or breast cancer [149,150,151,152]. Hypermethylation of hypoxia-inducible factor 3 Subunit Alpha (HIF-3α) in obese women has been associated with breast, oesophageal, thyroid and colorectal cancer [153]. Hypermethylation of SRGAP2C (Slit-Robo Rho GTPase activating protein 2C) and ZBTB46 (zinc finger and born-to-bind domain containing 46) results in tumorigenesis due to inhibition of their tumour suppressor gene function [146].

DNA methylation is implicated in 50–80% of colorectal cancers and is positively correlated with obesity and increasing age [146]. Hypermethylation in colorectal cancer is associated with CpG island hypermethylation of the Dickkopf (DKK) 1–3 genes and DKK-4 promoters and results in epigenetic silencing of their inhibition of canonical and non-canonical Wnt signalling pathways, and decreased expression of the de-methylating enzyme ten–eleven translocation methylcytosine dioxygenase 1 (TET1) [151]. Hypoxia can lead to a reduction in TET activity, leading to hypermethylation of gene promoters [154]. Furthermore, the hypomethylation of HIST1H3I (Histone linker 1 with Histone H3.1), HIST1H3D (Histone linker 1 with Histone H3.D), NFATC4 (Nuclear factor of activated T-cells cytoplasmic 4) and HOXB8 (Homeobox B8) and increase in their oncogene functions was associated with colon cancer and increased adiposity [146]. Apart from obesity increasing insulin, IGF-1, leptin, IL-6, TNF-α and decreasing levels of adiponectin which activates the PI3K-AKT signalling pathway in CRC, high BMI inhibits methylation of PI3K-AKT signalling pathway genes, constitutively activating the pathway [143].

### 9.2. Reversal of DNA Methylation

Recent studies have suggested that DNA methylation in morbid obesity may be reversible following bariatric surgery-induced weight loss and associated improvements in diet, metabolic profile, oxidative stress, ROS production and inflammatory markers [155,156]. The levels of genome-wide or genome-specific DNA methylation in patients after bariatric surgery were found to be similar to those of non-obese control patients. This was despite the postoperative BMI in patients still remaining in the Obesity Class I-II range (31.2–36.4 kg/m^2^), as compared to their preoperative Obesity Class III range (42.1–50.9 kg/m^2^) [155].

### 9.3. Butyrate and Epigenetic Suppression of CRC

Increasing the consumption of fermentable fibre can elevate the production of butyrate and promote its epigenetic role as a histone deacetylase inhibitor (HDACi), particularly in the colon. This is because normal colonocytes mainly use butyrate (70%) as a fatty acid energy source by *β*-oxidation in the mitochondria and there is little systemic absorption. However, neoplastic colonocytes which have undergone metabolic reprogramming via KRAS and the Warburg effect preferentially use glucose via cytosolic glycolysis. This means that more butyrate is transported to the colonocyte nucleus to act as an HDAC inhibitor. This potent tumour-suppressant effect of butyrate in colorectal cancer is dose-dependent, and thus reliant on healthy intestinal microbiota and adequate dietary intake of butyrogenic fibre. Other butyrate-associated epigenetic effects in CRC include suppression of host miRNA expression such as miR-17-92 cluster members by interfering with c-Myc; inhibition of HIF-1α and VEGF by promoting miR-199a; inhibition of CRC proliferation and invasion by promoting miR-203; and promotion of apoptosis by inhibition of NEDD9, whose overexpression activates the Wnt/ß-catenin signalling pathway [34,40,157].

## 10. Gut, Diet and Intestinal Microbiome

Exposure to exogenous and endogenously formed carcinogens through the consumption of highly processed foods, a high-fat diet, red meat and lack of dietary plant phytochemicals and antioxidants are implicated in carcinogenesis. The consumption of red meat, processed red meat preserved with sodium nitrite/nitrate and meat cooked at high temperature (grilling/frying/charbroiling) which produces nitrosamines, heterocyclic amines or advanced glycation end products is associated with obesity and gastric, kidney, pancreatic and colorectal cancer [22,33,42,158,159,160]. Heme from red meat may increase the endogenous generation of N-nitrosamines compared to white meat, and somatic mutations of KRAS and APC associated with CRC [154]. Elevated intestinal and plasma chymotrypsin levels associated with obesity and overeating have been correlated with increased insulin degradation, T2DM and cancer risk [100]. A Mediterranean diet contains dietary polyphenols, vitamins and omega-3 fatty acids derived from fish, fruits, nuts, vegetables and wholegrains, which have anti-inflammatory and anti-obesity effects [161]. A ‘green’ Mediterranean diet has higher amounts of dietary polyphenols which have been correlated with greater loss of VAT, in conjunction with lowered red meat intake and increased dietary fibre [33,46,52,159,161]. Phytochemicals including polyphenols, flavonoids, quinones, saponins and coumarins modulate the Wnt/*β*-catenin signalling pathway which inhibits transcription factors involved in adipogenesis such as C/EBPα and PPAR*γ*, preventing preadipocyte differentiation [158]. Clinical randomized controlled trials have shown that consumption of 50 g/day of dietary fibre reduced plasma glucose concentrations, daily urinary glucose excretion, 24 h plasma glucose and insulin concentrations, as well as blood cholesterol, triglyceride and very-low-density lipoprotein cholesterol [40].

### 10.1. Dietary Fibre

Increased dietary fibre intake, particularly from wholegrains, is associated with a decreased risk of obesity and CRC, as Denis Burkitt originally proposed [159,160,162]. Dietary fibre can be soluble or insoluble and includes:

1. Non-starch polysaccharides (NSP) from fruit and vegetables (cellulose, hemicellulose, pectin);
2. Non-digestible oligosaccharides (galactoligosaccharides, fructoligosaccharides, inulin);
3. Resistant starch from wholegrain cereals, root vegetables, nuts, seeds and legumes;
4. Lignins (non-carbohydrate phenolic polymers) [163].

The insoluble fibres cellulose, hemicellulose and lignin increase stool bulk, help retain water and reduce colonic transit time and mucosal exposure to carcinogens. Soluble NSPs such as *β*-glucans, pectins, guar gum and psyllium form a gel in the gut lumen which delays lipid and glucose absorption and the glycaemic response to food [163]. Dietary fibre and resistant starch that are not digested in the small intestine are fermented by anaerobic bacteria in the proximal colon. Such fermentation results in short-chain fatty acid (SCFA) production [164], which includes molar ratios of acetate 57: propionate 22: and butyrate 21 in the colonic lumen. Because butyrate is metabolized by colonocytes as their main microbial-derived nutritional source, the portal vein ratios of absorbed SCFAs (acetate 71: propionate 21: butyrate 8) differ from the colonic luminal concentrations. This regulates hepatic cholesterol synthesis, insulin sensitivity and lipoprotein metabolism. As propionate is metabolized by the liver, acetate becomes the major SCFA in the systemic circulation (acetate 91: propionate 5: butyrate 4), with effects on whole-body fat oxidation and energy expenditure [160].

### 10.2. Short-Chain Fatty Acids, Obesity and Cancer

Short-chain fatty acids maintain normal intestinal microbial diversity, gut mucus, colonocyte physiology and anti-tumour immunity [165]. Dysbiosis associated with a high-fat diet resulted in the decreased production of bacterial SCFAs and promoted G12D mutant Kras-driven intestinal carcinogenesis in a murine serrated hyperplasia model. Butyrate was shown to reverse this process [15]. Resistant starch is highly butyrogenic and in controlled human studies inhibited colorectal mucosal proliferation markers, oncogenic miRNA (miR17–92) and adenomatous colonic polyp formation [40]. Butyrate has been shown to suppress colonic oncogene signalling (Wnt, SMAD3, MAPK1) [166]. SCFAs bind to colonic G-protein coupled receptors (GPCR), also known as free fatty acid receptors (FFAR) and suppress colonic mucosal proliferation by stimulating IL-10, FOXP3 expression and regulatory T cells. Butyrate has also been shown to inhibit glycolysis in colorectal cancers by a reduction in glucose transport (GLUT-1) and cytoplasmic G6P via the GPCR109a-AKT signalling pathway [167]. Activation of intestinal FFARs stimulates the release of glucagon-like peptide-1 (GLP-1) and peptide YY (PYY) from enteroendocrine cells, which regulates host metabolism signalling, hypothalamic orexigenic neurons expressing NPY/AgRP, ghrelin signalling, glucose stimulated-insulin secretion and leptin release. Circulating acetate can also cross the blood–brain barrier and directly activate hypothalamic FFARs to suppress appetite and decrease energy intake, as well as increase thermogenesis and fat oxidation. Sources of acetate include vinegar-containing foods or colonic saccharolytic fermentation of non-digestible oligosaccharides such as unprocessed wheat bran [40,160]. Up to 10% of all human energy expenditure can be provided by microbial SCFAs after absorption from the colon. Microbial generation of SCFAs thus maintains normal circadian cycles of host energy metabolism, BAT activation and appetite/satiety and is protective against the development of obesity and CRC [168].

Although fermentation of amino acids contributes less than 1% of the microbial derived intestinal SCFAs, in the carbohydrate-depleted distal colon, proteolytic fermentation by specialist bacteria leads to the release of toxic metabolites including ammonia, phenols and sulphides. This may increase the risk of rectosigmoid CRC in high-red meat, low-fibre diets [15,163,169,170,171,172,173]. Maintenance of normal gut mucosal integrity and cellular tight gap junctions is important in minimizing absorption and portal circulation of harmful gut-derived metabolites (e.g., microbial LPS, bile acids) which are implicated in NASH, HCC and T2DM [101,144,162,173]. Other dietary protective factors are Bowman-Birk protease inhibitors, which are heat-resistant plant proteins in legumes and cereals. They may prevent colorectal carcinogenesis due to their ability to inhibit the down-regulation of tumour suppressors by serine proteases (e.g., trypsin/chymotrypsin) [100].

### 10.3. Obesity and Intestinal Estrolobiome

Western-type high-fat, low-fibre diets associated with obesity change the intestinal microbiome, which can alter the hepatic excretion, metabolism and intestinal reabsorption of steroid molecules such as oestrogens and bile acids [174,175]. Dysbiosis of the gut microbiome can lead to the deconjugation of conjugated oestrogens (E2, oestrone) by gut microbial glucuronidase (GUS) enzymes, part of the gut estrolobiome [164]. This increases the levels of free oestrogens that can be reabsorbed by the enterohepatic circulation, elevating systemic oestrogen levels and increasing the risk of oestrogen-sensitive cancers expressing ER-α/GPER. In contrast, plant-based diets or low-saturated-fat diets are associated with increased faecal levels of oestrogens and lower plasma oestrogen levels [176,177]. Some studies revealed altered composition and reduced faecal microbiome diversity in women with breast cancer [174,175]. Others concluded that the doubling in urinary oestrogen levels in postmenopausal women diagnosed with breast cancer compared to control subjects with normal mammography was independent of these gut microbiome changes [178]. The estrolobiome is more efficient in females than males, which is related to sex differences in oestrogen cycling and gut microbiome diversity [177].

Phyto-oestrogens are conjugated with glucose in plants as inactive glycosides. Major sources of phyto-oestrogens include isoflavones from legumes (e.g, genistein, daidzein); lignans from flaxseed, lentils, whole grains, beans (enterodiol, enterolactone; coumestans from brussel sprouts, spinach, legumes (coumestrol); stilbenes from peanuts, berries, grapes and red wine (resveratrol); flavanones from citrus fruit (quercetin); flavonols from green tea (catechins); and flavones from herbs and cereals (apigenin, luteolin). After ingestion, dietary phyto-oestrogens are hydrolysed by gut bacterial UDP-glucuronosyltransferase to their active forms and readily absorbed. As compared to E2, dietary phyto-oestrogens generally bind with higher affinity to the ER-*β* and lower affinity to the ER-α. ER-*β* is the predominant estrogen receptor in normal colonic mucosa in both males and females, and progressive loss of ER-*β* expression is correlated with increased mucosal inflammation, adenomatous polyp formation and colorectal carcinogenesis. Activation of ER-*β* may be protective against the development of CRC, breast, ovarian, prostate and hepatocellular adenocarcinoma. This is also dependent on the ratio of ER-α/ER-*β* expression in different tissues. Activation of ER-*β* regulates the ER-α and prevents unopposed ER-α activation and cellular proliferation. Dietary phyto-oestrogens can thus act as selective estrogen receptor modulators (SERMs) [104,177].

### 10.4. High-Fat Diet, Bile Acids and Cancer

Elevation of faecal secondary bile acids (deoxycholic acid/lithocholic acid) due to high-fat/meat-based diets is thought to lead to increased colonic mucosal inflammation and permeability, *cox-2* expression and somatic mutations in tumour suppressor genes (*p53*) or APC regulator of WNT signalling pathway gene (*APC*), thereby promoting the development of sporadic CRC [179]. A plant-based high-fibre diet is thought to bind bile acids and decrease their absorption in the terminal ileum and formation of secondary bile acids from primary bile acids. In contrast, Western-style diets high in animal protein and fat (meat, eggs, cheese) and low in dietary fibre expand the bile acid pool, faecal levels of total and secondary bile acids and faecal anaerobic microbial pathogens [173]. The increased entero-hepatic circulation of deoxycholic acid (a secondary bile acid metabolite of *Clostridium* spp.) has been implicated in the development of HCC, by inducing a SASP in hepatic stellate cells. Apart from being identified as an endogenous carcinogen in HCC, CRC, biliary tract, gall bladder and Barrett’s oesophageal adenocarcinoma, deoxycholic acid has also been implicated as a carcinogen in breast cancer [180,181]. Plasma levels of deoxycholic acid were 52% higher in breast cancer patients than the control patients. *Clostridium XIV* have been isolated from both the breast and gut microbiome of patients with breast cancer. The *Clostridium XIV* specific deoxycholic acid metabolite was shown to stimulate proliferation in HER-2 positive breast cancer cells but not TNB cancer cells [182].

## 11. Weight loss and Prevention of Cancer

Exercise may prevent some of the adverse effects of a high-fat Western diet, including fat deposition, glucose intolerance and leptin release. Maintaining a lean WAT phenotype stimulates adiponectin release, which activates AMPK and blocks ROS-mediated pathways, including oxidized LDL, lectin-type oxLDL receptor (LOX-1)/NF-κB activation and the formation of foamy macrophages [178,179,183,184,185,186,187,188,189,190,191,192,193,194,195,196,197,198,199,200,201,202,203]. This prevents atherosclerosis, cellular senescence and the development of cancers driven by hypersecretion of inflammatory adipokines, including leptin. Adiponectin was also shown to protect pancreatic islet *β*-cells from glucolipotoxicity and promoted insulin secretion in response to glucose loading and normalized pancreatic cellular respiration and oxygen consumption. Adiponectin, therefore, antagonizes the leptin-induced stimulation of glucose transport, glycolysis, oncogenic signalling and the Warburg effect [204,205,206] (Figure 5). However, when an individual develops morbid obesity and loss of adiponectin secretion, significant weight loss is unlikely to be achieved by conventional means (diet/exercise/counselling) without pharmacological or bariatric surgical intervention [197]. This is related to the consumption of obesogenic Western diets, pathological VAT accumulation, disrupted homeostasis, metabolic syndrome and persistent inflammatory, neurohormonal and exosomal signalling pathways [55,183,184,185,186,187,188,189,190,191,192,193,194,207,208,209].

The failure of conventional obesity treatment was clearly demonstrated in the Swedish Obesity Subjects (SOS) study, a randomly selected prospective bariatric surgery intervention trial. The SOS study reported that after 20 years follow-up, bariatric surgical patients had a mean TBW loss of 18% (22.1 ± 16.5 kg) as compared to 1% in control patients [195]. Laparoscopic sleeve gastrectomy (LSG) and Roux-en-Y gastric bypass (RYGB) are classified as bariatric metabolic surgeries (BMS), as compared to restrictive bariatric procedures such as laparoscopic adjustable gastric banding (LAGB) or vertical banded gastroplasty (VBG). The improvement in insulin sensitivity (HOMA-IR) after RYGB begins as early as one week after surgery. This is related to the acute alterations in GLP-1, peptide YY, cholecystokinin, oxyntomodulin, and leptin, even before weight loss becomes a feature. Falls in inflammatory markers (TNF-α, hs-CRP, IL-12, IL-6, and IFN- α2) occur later at 3–6 months post RYGB, which correlate with decreased triglyceride levels, body weight, fat mass, BMI and body-fat percentage [194,196].

The improvements in insulin sensitivity and systemic inflammation after BMS may be transient if patients do not substantially change their diets or increase their exercise level. A combination of resistance and aerobic exercise enhances the improvements in endothelial function and prevents sarcopenia and bone loss after BMS. The sustained improvement in muscle mass and strength with exercise after BMS is related to changes in the transcriptome, including suppression of the TGF-*β*1/SMAD-2/3 ECM pathway and promotion of follistatin. This does not occur with BMS alone. Skeletal muscle hypertrophy helps to maintain insulin sensitivity and the long-term remission of T2DM after BMS [183]. Consumption of sweets, binge or emotional eating, large portion size and lack of whole fruit consumption are significant dietary risk factors for weight regain, with one in six patients having ≥ 10% weight regain at a mean follow-up of 5.2 years after BMS [187].

Meta-analyses of pooled studies have suggested bariatric surgery results in a substantial improvement in the future risk of oestrogen-sensitive cancers in women (breast, endometrial, ovarian); overall cancer incidence; non-hormonal obesity-associated cancers; and cancer-related mortality [189,190,197,209]. This is related to the reversal of adipocyte dysfunction, OSA, NASH, hyperlipidaemia, aromatase activity, systemic inflammation, hyperinsulinaemia, and metabolic syndrome with the loss of VAT volume and decreased CLS/WAT inflammation after BMS [61,198,199,200,201,202,203]. Changes in food choices, carcinogen ingestion, VAT exosome release, intestinal dysbiosis and exercise capacity may also contribute to the prevention of future cancers after BMS.

**Figure 11:**
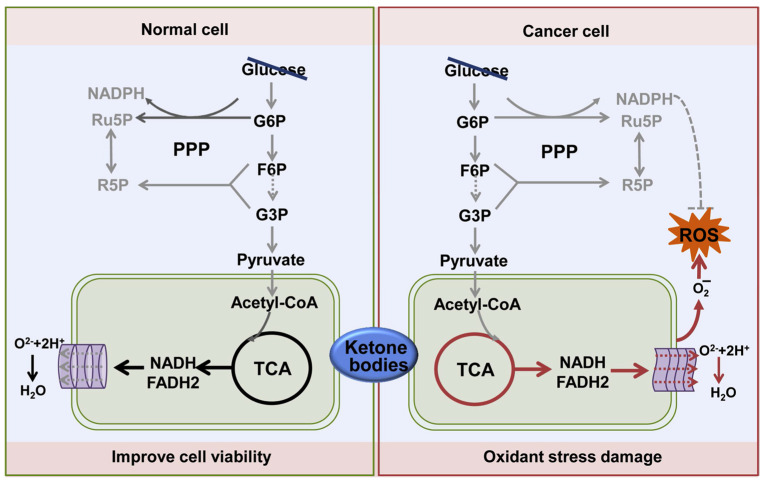
The different regulation of ketone bodies between normal and cancer cells under low-carbohydrate conditions. Normal cells can catabolize ketone bodies into acetyl-CoA, which enters the TCA cycle to produce energy and improve cell viability under low-carbohydrate condition. However, excessive electrons produced by NADH/FADH2 in enhanced TCA cycle are transported to the mitochondrial respiratory chain in cancer cells, which generate ROS. In addition, the antioxidant system (PPP) pathway is inhibited under low-carbohydrate conditions, which results in excessive ROS production and leads to oxidative stress damage. Reprinted with permission from [217]. Copyright © 2023 Elsevier.

There is limited clinical data on the influence of very-low-calorie, ketogenic (VLCK) diets or vegetarian diets on cancer mortality, with some evidence of high adherence to a Mediterranean diet improving cancer incidence and cancer-related mortality [210,211,212,213,214,215]. Some cancers have decreased capacity to feed ketone bodies such as *β* -hydroxy butyrate and acetoacetate into the TCA cycle, as compared to normal tissues. This is due to deficiencies in succinyl-CoA:3-ketoacid coenzyme A transferase (OXCT1/SCOT), 3-hydroxybutyrate dehydrogenase 1 (BDH1) and acetoacetyl-CoA thiolase in cancer cell mitochondria. The ‘traditional’ very-low-calorie, high-fat ketogenic diet consists of 90% fat, 8% protein and 2% carbohydrates. Such diets mimic a fasting state, generate large amounts of ketone bodies and minimize the flux of glucose down the PPP. This prevents the production of NADPH and thus may increase ROS in susceptible cancer cells. Ketogenic diets may be synergistic with other therapies such as the glycolysis inhibitor 2-deoxy-D-glucose, PD-1 blockade or chemoradiotherapy in tumours including malignant glioma, pancreatic, lung, prostate, colorectal or breast cancer. However, the promising results of ketogenic diets in animal studies and early clinical case series have been difficult to demonstrate in recent systematic reviews and meta-analyses of human trials. Sufficiently powered, randomized controlled trials are required to prove the benefits [216,217,218,219,220] (Figure 11).

Obesity and metabolic syndrome not only increase the risk of primary cancers, but also influence prognosis after cancer treatment [221,222,223,224,225]. Lower cancer recurrence rates and improved survivorship are reported in bariatric surgical patients who were subsequently treated for cancer, compared to obese control patients [197,226,227,228]. The effects of bariatric surgery appear to be ‘dose dependent’, with a TBW loss 12 months after BMS of >30% having the greatest beneficial effect, and <20% TBW loss having the poorest effect on cancer-free survival at 10 years follow-up [185]. In a recent meta-analysis of bariatric surgery and non-hormonal cancers including liver, colorectal, kidney, oesophageal and lung cancer, both LSG and RYGB were associated with a future decrease in overall cancer risk, but LAGB was not. In particular, the future risk of CRC after bariatric surgery compared to obese control patients was significantly less after RYGB (OR = 0.47, 95% CI, 0.36– 0.61, I2 = 64.22%) and LSG (OR = 0.55, 95% CI, 0.36–0.83, I2 = 77.81%), but not after LAGB (OR = 1.34, 95% CI, 0.28–7.12, I^2^ = 99.08%) [189].

## 12. Summary and Conclusions

In the 1970s, Denis Burkitt proposed that an ingestion threshold of 50 g per day of dietary fibre was necessary to inhibit cholesterol absorption, improve constipation and straining at stool, and prevent Western diet-related diseases including obesity, diabetes, hyperlipidaemia, hypertension, atherosclerotic cardiovascular disease, colonic diverticular disease and colorectal cancer. Increased consumption and absorption of saturated fats and highly processed carbohydrates with virtually no dietary fibre contribute to the metabolic syndrome and to subsequent obesity-related carcinogenesis. This is mediated by the deposition of triglycerides in white adipose tissue (VAT > SAT) and cholesterol in hepatocytes. The subsequent inflammatory response results in the formation of crown-like structures and a dysfunctional SASP. The release of prostaglandins, cytokines, oestrogens, adipokines and exosomes from CLSs is fundamental to the pathogenesis of obesity and obesity-associated cancers. Hyperinsulinaemia, leptin, aromatase activation and 17-*β*-oestradiol synthesis in WAT CLS drive the increased incidence of oestrogen-sensitive cancers in women with obesity (postmenopausal breast cancer, endometrial cancer, ovarian, thyroid cancer).

The molecular mechanisms involved in the genesis of obesity are intricately related to the hallmarks of cancer. These include sustained proliferative signalling, EMT, epigenetic effects, metabolic reprogramming, somatic gene mutations, intestinal dysbiosis, cellular senescence, evasion of growth suppressors and angiogenesis. The increasing prevalence of overweight and obesity in the global population stems from rapid urbanization; mass production and distribution of highly processed food; overconsumption of energy and sedentary lifestyles; and is correlated with increasing rates of obesity-associated cancers in both developed and developing countries.

## Abbreviation

17β-HSD	17β-hydroxysteroid dehydrogenase
AdipoR1/AdipoR2	adiponectin membrane receptors
ADM	adrenomedullin
A-FABP	adipose fatty acid binding protein
AgRP	agouti-related peptide
AHI	apnoea-hypopnoea index
AKT	Protein kinase B (PKB)
AMPK	AMP-activated protein kinase
ANGPTL4	angiopoietin-like 4
*APC*	adenomatous polyposis coli regulator of WNT signaling pathway gene
ATM	adipocyte tissue macrophages
ATP	adenosine triphosphate
β-HB	beta-hydroxy butyrate
BAT	brown adipose tissue
BCL-2	B cell lymphoma 2
BMI	body mass index
BMS	bariatric metabolic surgery
BRAF	v-raf murine sarcoma viral oncogene homolog B1
CAA	cancer-associated adipocytes
CAF	cancer-associated fibroblasts
Cav-1	caveolin-1 structural protein
CCAAT	cytosine-cytosine-adenosine-adenosine-thymidine
CCK	cholecystokinin
CCL-2	Chemokine (C-C motif) ligand 2, MCP-1
CD36	Cluster of differentiation 36
CDKN1a	Cyclin Dependent Kinase Inhibitor 1A
CI	confidence interval
CIMP	CpG dinucleotide island methylator phenotype
CLS	crown-like structures
CMP-1	chemoattractant monocyte protein 1
CMS	consensus molecular subtype
c-Myc	cellular myelocytomatosis
COX-2	cyclooxygenase
CpG	cytosine and guanine separated by a phosphate
CRC	colorectal cancer
CRP	C-reactive protein
c-Src	cellular analogue of Rous sarcoma virus
CXCL-1	C-X-C Motif Chemokine Ligand 1
CYP19A1	cytochrome P450 19A1
DAMPS	damage-associated molecular patterns
*db*/*db*	Homozygous mutation in murine leptin receptor gene
DDR	DNA damage response
DKK-3	Dickkopf 3 protein
DMR	Differentially methylated regions
E1	oestrone
E2	17-β-oestradiol
EBPα	enhancer-binding proteins α
ECM	extracellular matrix
EGFR	epidermal growth factor receptor
EMT	epithelial to mesenchymal transition
END	endurance
EPO	erythropoietin
ER-a	oestrogen receptor alpha
ERK	Extracellular Signal-Regulated Kinases
ESCRT	endosomal sorting complexes required for transport
FAO	Fatty acid oxidase
FasL	FS-7-associated surface antigen ligand
FFA	free fatty acid
G-6-PD	glucose 6-phosphate dehydrogenase
GAPDH	glyceraldehyde-3-phosphate dehydrogenase
G-CSF	granulocyte-colony stimulating factor
GIP	glucose dependent insulin peptide
GLP-1	glucagon like peptide
GLUT-1 and -3 and -4	glucose transporters
GPCR	G-protein coupled receptor
GPER	G-protein-coupled oestrogen receptor
GSH	glutathione
GUS	glucuronidase enzymes
HCC	hepatocellular carcinoma
HDACi	histone deacetylase inhibitor
HDL	high-density lipoprotein
HER2	human epidermal growth factor receptor
HFD	high-fat diet
HGF	hepatocyte growth factor
HIF	hypoxia-inducible factor
HIF-1α	hypoxia-inducible factor-1α
HIIT	high intensity interval training
HIST1H3D	Histone linker 1 with Histone H3.D
HIST1H3I	Histone linker 1 with Histone H3.1
HK-1	hexokinase 1
HK2	hexokinase 2
HOMA-IR	Homeostatic Model Assessment of Insulin Resistance
HOXB8	homeobox B8
HRE	hypoxia-response element
HSP	heat shock protein
HSP-90	heat shock protein 90
ICAM	intercellular adhesion molecule
ID2	DNA-binding protein inhibitor 2
IGF-1	insulin-like growth factor 1
IGF-1R	intracellular insulin receptor substrate 1
IGF-2	insulin-like growth factor 2
IGF-BP	IGF binding proteins
IL-1	Interleukin 1
IL-10	Interleukin 10
IL-1β	Interleukin 1β
IL-6	interleukin 6
IL-8	interleukin 8
ILK	Integrin-linked kinase
INF-γ	interferon gamma
iNOS	inducible nitric oxide synthase
IRS	insulin receptor substrate
JAK2	Janus kinase 2
JMJD1A/JMJD2B	jumonji family histone demethylase
KRAS	Kirsten rat sarcoma viral oncogene homolog
LDHA	lactate dehydrogenase A
LDL	low density lipoprotein
LEPR	Long isoform of neural leptin receptor
LPA	lysophosphatidic acid
LSG	sleeve gastrectomy
LPS	lipopolysaccharide
MAC-2	macrophage-2 binding protein, galectin-3
MAPK	mitogen-activated protein kinase
MC4R	melanocortin 4 receptor
MCP-1	monocyte chemoattractant protein 1
MCT-1/MCT-4	mono-carboxylate transporter
Mdm2	mouse double minute 2 homolog
MetS	metabolic syndrome
MHO	metabolically healthy obesity
MIP-2	macrophage inflammatory protein, CXCL2
MMe	metabolically activated macrophage
MMP	mixed metalloproteases
MRAP2	Melanocortin 2 Receptor Accessory Protein 2
MSI	microsatellite instability
mTOR	mammalian target of rapamycin
MUNW	metabolically unhealthy–normal weight
NADPH	Reduced nicotinamide adenine dinucleotide phosphate
NAFLD	non-alcoholic fatty liver disease
NASH	non-alcoholic steatohepatitis
NFATC4	nuclear factor of activated T-cells cytoplasmic 4
NF-*κ*B	nuclear factor kappa light chain enhancer of activated B cells
NLRC4	nucleotide-binding domain and leucine-rich repeat receptor family CARD domain containing 4
NO	nitric oxide
NOS	nitric oxide synthase
NOX	NADPH oxidase
NPY	neuropeptide Y
OAC	obesity-associated cancers
Ob-Rb	long form leptin receptor
OCT4	octamer-binding transcription factor 4
OHS	obesity hypoventilation syndrome
OR	odds ratio
OSA	obstructive sleep apnoea
PAI-1	plasminogen activator inhibitor-1
PCSK1	proprotein convertase subtilisin/kexin type 1
PDGF-BB	platelet-derived growth factor-BB
PDH	pyruvate dehydrogenase
PDHK	pyruvate dehydrogenase kinase
PFK1	phosphofructokinase
PFKFB3	6-phosphofructo-2-kinase/fructose-2, 6-bisphosphatase 3
PGE2	Prostaglandin E2
PGK-1	phosphoglycerate kinase 1
PHD2	prolyl hydroxylase domain containing 2
PHIP98	Pleckstrin Homology Domain Interacting Protein 98
PI3K	phosphoinositide 3-kinase
PLCγ-PKC	phospholipase C (PLC)γ-protein kinase C (PKC)
PKM2	pyruvate kinase muscle 2
POMC	pro-opiomelanocortin
PPARγ	peroxisome proliferator-activated receptor γ
PPP	pentose phosphate pathway
pVHL-VBC	von Hippel-Lindau protein (pVHL)-elonginB-elonginC (VBC) complex
Rab	Ras-associated binding proteins
RAF	rapidly accelerated fibrosarcoma
RAS	rat sarcoma
RhoA-ROCK	Ras homology gene family member A—Rho-associated kinases
RNA: lncRNA	long non-coding RNA
RNA: miRNA	micro-RNA
RNA: mRNA	functional RNA
RNA: snRNA	small nuclear RNA
RNA: tRNA	transfer RNA
ROS	reactive oxygen species
RYGB	Roux-en-Y gastric bypass
SASP	senescence-associated secretory phenotype
SAT	subcutaneous adipose tissue
SCFA	short-chain fatty acids
SFRP5	secreted frizzled-related protein 5
SH2B1	Src-homology 2B adaptor protein 1 gene
SHBG	sex hormone-binding globulin
SIM1	Single-minded 1
SMAD3	mothers against decapentaplegic homolog 3
SOX2	SRY-Box Transcription Factor 2
SRGAP2C	Slit-Robo Rho GTPase activating protein 2C
STAT3	Signal transducer and activator of transcription 3
T2DM	Type II diabetes mellitus
TBW	total body weight
TCA	tricarboxylic acid
TET	ten–eleven translocation methylcytosine dioxygenase
TfR	transferrin receptor
TGF-α	transforming growth factor α
TGF-β	transforming growth factor β
TIMP	tissue inhibitor of metalloproteinase
TKR	tyrosine kinase receptor
TLR	Toll-like receptor
TNBC	triple negative breast cancer
TNF-α	tumor necrosis factor alpha
TP53	tumor suppressor protein 53
TPI1	triosephosphate isomerase
TRAIL	tumor necrosis factor-alpha-related apoptosis inducing ligand
UCP-1	uncoupling protein 1
VAT	visceral adipose tissue
VEGF	vascular endothelial growth factor
WAT	white adipose tissue
Wnt	wingless-related integration site
XOX	xanthine oxidase
ZBTB46	zinc finger and born-to-bind domain containing 46
